# Pyridine-Based Multifunctional Surface Passivators Enable Efficient and Stable Perovskite Indoor Photovoltaics

**DOI:** 10.1021/acsami.5c08539

**Published:** 2025-08-21

**Authors:** Yi Han, Ceylan Doyranli, Alessia Di Vito, Matthias Auf der Maur, Mokurala Krishnaiah, Paavo Mäkinen, Ramesh Kumar, Basheer Al-Anesi, Debjit Manna, Paola Vivo

**Affiliations:** † Hybrid Solar Cells, Faculty of Engineering and Natural Sciences, 7840 Tampere University, Tampere FI-33014, Finland; ‡ Department of Electronic Engineering, 9318 University of Tor Vergata, Rome 00133, Italy; § Department of Chemistry − Ångström Laboratory, 8097 Uppsala University, Uppsala SE-75120, Sweden

**Keywords:** Indoor Photovoltaics, Surface Passivation, Pyridine, Defect Suppression, Perovskite Solar Cells

## Abstract

Efficient surface passivation is crucial for mitigating defect-induced recombination losses in perovskite-based indoor photovoltaics (IPVs), where charge carrier dynamics are particularly sensitive to trap states under low-intensity illumination. Here, we introduce two pyridine-based passivators, tris­[4-(pyridin-4-yl)­phenyl]­amine (**TPAP**) and its ionic counterpart (**TPAP1**), to achieve high-performance and stable perovskite IPVs. These passivators strongly coordinate with under-coordinated Pb^2+^ ions, effectively reducing trap densities and improving hydrophobicity. When incorporated into lead-based triple-cation CsFAMA perovskite films, **TPAP** and **TPAP1** significantly suppress nonradiative recombination, leading to notable improvements in device performance. Remarkably, **TPAP1** demonstrates a unique ability to simultaneously passivate multiple defect types, further optimizing charge transport and boosting the open-circuit voltage (*V*_OC_). As a result, IPV devices incorporating **TPAP** and **TPAP1** achieved remarkable indoor power conversion efficiencies of 30.1% and 31.7%, with *V*_OC_ values of 0.97 and 1.00 V, respectively, under 1000 lux white LED illumination. This study presents a scalable and effective strategy for defect passivation in perovskite IPVs, highlighting the critical role of multifunctional organic passivators in advancing next-generation energy harvesting technologies.

## Graphical abstract

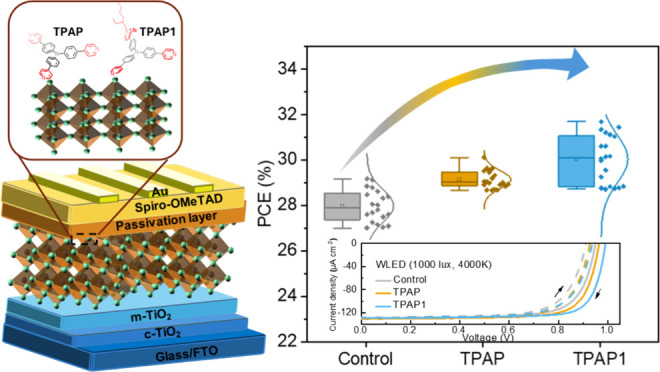

## 1 Introduction

Rapid progress in Internet of Things (IoT) technologies has sharply increased the need for efficient indoor energy harvesting.^1,2^ Among various approaches, indoor photovoltaics (IPVs) have gained attention as a promising means to power IoT devices by converting low-intensity ambient light, typically emitted from artificial sources such as LED or fluorescent lamps, into electrical energy.^3–5^ Unlike outdoor solar cells that operate under direct sunlight, IPVs must demonstrate high efficiency under low-intensity light conditions, which poses distinct challenges in terms of material performance and device design.^6^

Perovskite solar cells (PSCs) are attracting increasing attention for IPV applications, owing to their favorable optoelectronic characteristics, including tunable bandgaps, high-power conversion efficiency (PCE) efficient light harvesting, and low-cost fabrication processes.^7,8^ Their wide absorption range and tunable bandgap, which can be tailored to align with the emission spectra of typical indoor light sources, make these devices highly suitable for indoor applications.^9,10^ Despite their promise, achieving high-performance, stable PSCs under low-light indoor conditions remains a formidable challenge.^11,12^ A key issue is the presence of structural defects in perovskite films, particularly at grain boundaries and surfaces, which introduce trap states that facilitate nonradiative charge recombination.^13–15^ These defects significantly limit photovoltaic performance—especially open-circuit voltage (*V*_OC_) and fill factor (FF)—and accelerate long-term device degradation.^16,17^ Such defects commonly originate from incomplete coordination of lead (Pb^2+^) ions, unsatisfied bonds at the surface, and impurities introduced during film deposition processes.^15,18^ The negative impact of these defects is exacerbated under the low-intensity light typical of indoor environments, where photogenerated charge carriers are more susceptible to trap-assisted recombination due to their higher sensitivity to defect density compared to outdoor illumination.^19,20^ To mitigate this issue, Jie et al. introduced EuCl_3_ into triple-cation perovskite films, achieving improved film crystallinity and reduced carrier recombination under indoor lighting. Using the optimized EuCl_3_-doped formulation, they reported a notable indoor PCE of 30.0% and a *V*_OC_ of 0.91 V under 1000 lux.^21^ Moreover, our group investigated how doping Spiro-OMeTAD influences the performance of triple-cation lead halide perovskite devices under both 1 sun and indoor white LED (WLED) lighting conditions. Under 1000 lux, 4000 K lighting conditions, the doped-Spiro-OMeTAD devices achieved a PCE of 29.1% and a *V*_OC_ of 0.95 V, outperforming their undoped counterparts, particularly at low-light intensities.^22^ However, despite these encouraging results, further enhancement remains essential for approaching the theoretical limits of indoor photovoltaic performance.^23,24^ Therefore, addressing these defects is essential to simultaneously achieving high efficiency and long-term stability in perovskite-based IPVs.

To address these challenges, surface passivation strategies have gained widespread attention as an effective approach to suppress defect-induced recombination.^25,26^ The presence of surface traps severely limits charge carrier transport across the perovskite/HTL interface, which lowers extraction efficiency and degrades overall photovoltaic performance.^25,27^ In these devices, the strong coupling between mobile ions and charge carriers, particularly near the cathode, can lead to interfacial recombination losses.^28^ Reducing this recombination and minimizing electron–ion coupling are crucial for improving the performance of IPVs. The primary role of surface passivators is to eliminate interfacial defects, thereby suppressing nonradiative recombination and enhancing photovoltaic output. For example, Gratzel’s group used electron-withdrawing molecules to passivate the surface defects and improve the interface dipole effect, improving the device performance up to 25.21%.^29^ Wong’s group introduced coplanar heteroacene cored A–D–A-type molecules to modify the perovskite surface, improving passivation and interfacial contact, thereby achieving highly efficient and stable PSCs.^30^ Among the various chemical agents explored for surface passivation, nitrogen-containing heterocyclic compounds, such as pyridine and its derivatives, have demonstrated exceptional effectiveness.^31–33^ These compounds interact strongly with under-coordinated Pb^2+^ ions in the perovskite lattice, forming stable complexes that neutralize defects and reduce surface trap densities.^31^ The lone electron pair located on pyridine’s nitrogen atom is readily donated to the empty orbitals of Pb^2+^, thereby reducing recombination losses and facilitating more efficient charge extraction.^31,34^ In most cases, conventional additives or passivators are limited to targeting either positively or negatively charged defects. However, perovskite materials can host a diverse range of defect types, including antisite defects like PbI_3_^–^, under-coordinated halide ions and Pb^2+^, as well as iodine vacancies.^35^ Therefore, developing a passivator capable of addressing multiple defect types simultaneously is highly desirable, particularly for indoor photovoltaics, where effective defect passivation becomes even more critical due to the lower photon flux.^20^

In this study, we introduce two pyridine-based passivators, tris­[4-(pyridin-4-yl)­phenyl]­amine (**TPAP**) and its ionic counterpart (**TPAP1**), designed to boost both efficiency and long-term stability in perovskite indoor photovoltaics. The molecular design combines three key features: (1) multiple pyridine groups for strong Lewis base coordination with undercoordinated Pb^2+^, (2) a triphenylamine core and aromatic pyridine substituents for improved moisture resistance, and (3) in **TPAP1**, selective quaternization of one pyridine unit creating a hybrid neutral/ionic structure. This unique design enables **TPAP1** to simultaneously passivate both positively charged Pb^2+^ (via pyridine coordination) and negatively charged halide vacancies (via pyridinium electrostatic interaction), a capability beyond conventional single-mechanism passivators. Both **TPAP** and **TPAP1** were incorporated into lead-based triple-cation CsFAMA perovskite (Cs_0.05_(MA_0.17_FA_0.83_)_0.95_Pb­(I_0.83_Br_0.17_)_3_) films, a composition widely recognized for its balanced optoelectronic properties.^36^ The addition of these passivators effectively reduced trap densities and improved the hydrophobicity of the perovskite film, leading to enhanced stability and efficiency under typical low-light indoor conditions. Importantly, the ionic **TPAP1** exhibited an exceptional ability to simultaneously passivate multiple types of defects. As a result, IPV devices incorporating **TPAP** and **TPAP1** achieved remarkable PCEs­(i) of 30.1% and 31.7%, with *V*_OC_ values of 0.97 and 1.00 V, respectively, utilizing a perovskite absorber layer with a band gap of approximately 1.60 eV, demonstrating the potential of pyridine-based passivation strategies for high-performance IPVs. By offering a scalable approach to performance enhancement without requiring complex fabrication processes, this work advances defect passivation techniques for indoor photovoltaics and highlights the role of organic passivators with a strong lead ion affinity in unlocking the potential of perovskite materials for indoor applications.

## 2 Results and Discussion

### 2.1 Basic Properties

The molecular structures of **TPAP** and **TPAP1**, shown in Figure 1a, were designed as triphenylamine-core derivatives with pyridine functional groups to enable specific coordination or electrostatic interactions for passivating perovskite defects. The synthetic pathways for **TPAP** and **TPAP1** are illustrated in Figure 1a, with experimental details provided in Section 4 and characterization data included in Figures S1–S3. **TPAP** was synthesized via a Suzuki coupling reaction to introduce pyridine groups onto the triphenylamine core. Subsequently, **TPAP** undergoes quaternization with 2-ethylhexyl bromide to form the ionic derivative **TPAP1**.^37^ This synthetic route effectively incorporates pyridine groups into the triphenylamine core and enables further ionic modification. Remarkably, **TPAP** can be synthesized in a single step, while **TPAP1** is obtained in just two steps, demonstrating the high synthesis efficiency and simplicity of both passivators. The thermal, optical, and electrochemical characteristics of the synthesized passivators can be found in Table 1 and the obtained measurement results are shown in Figure S4 (thermogravimetric analysis), Figure S5 (ultraviolet–visible absorption spectra), Figure S6 (fluorescence spectra), and Figure S7 (cyclic voltammograms).

**Figure 1:**
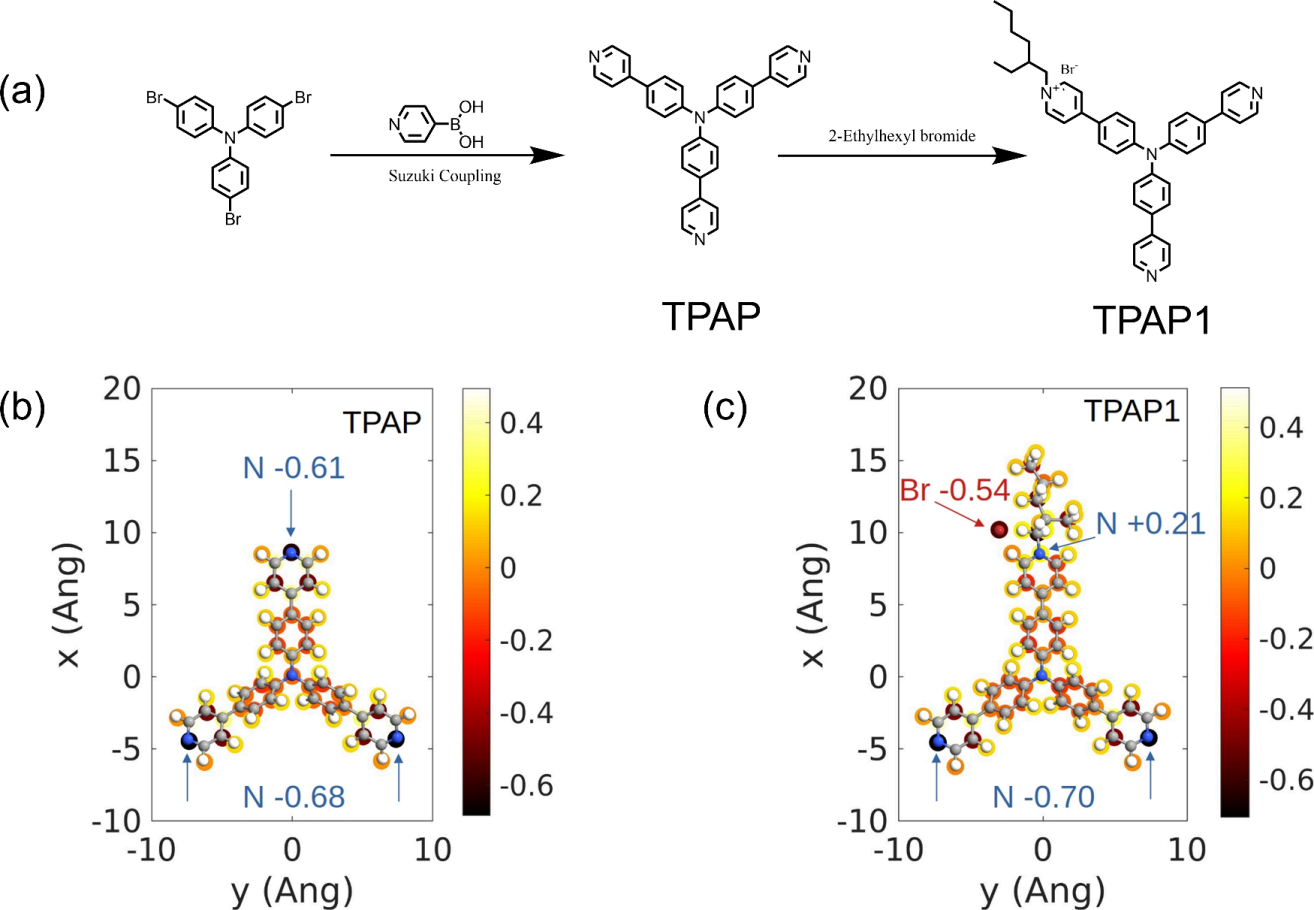
(a) Synthetic route of **TPAP** and **TPAP1**. Atomic partial charges were derived by ESP analysis for (b) **TPAP** and (c) **TPAP1** in their optimized configuration, where N, C, H, and Br atoms are represented in blue, gray, white, and red, respectively. The color bar indicates the magnitude of the atomic partial charges in units of *e*.

**Table 1:** Thermal, Optical, and Electrochemical Characteristics of the Synthesized Passivators

passivator	*T*_dec_ [°C]^a^	λ_max_ [nm]^b^	*E*_g_ [eV]^c^	HOMO_exp_ [eV]^d^	LUMO_exp_ [eV]^e^
**TPAP**	395	397	3.12	–5.75	–2.58
**TPAP1**	309	483	2.56	–5.70	–3.19

a Decomposition (T_dec_, 5% weight loss) recorded by TGA measurements (10 °C/min, N_2_ atmosphere).

b Maximum absorption values determined in DMF solution.

c *E*_g_ = 1240/λ_onset_ (eV).

d Calculated from cyclic voltammetry using the equation: HOMO_exp_ = −[*E*_ox_ – *E*(Fc/Fc^+^) + 5.10] (eV).

e LUMO_exp_ = HOMO_exp_ + *E*_g_ (eV).

The ultraviolet–visible absorption spectra (UV–vis) of **TPAP** and **TPAP1** in DMF are shown in Figure S5a, with their maximum absorption peaks and onset wavelengths (λ_max_/λ_onset_) recorded at 357/397 and 421/483 nm, respectively. Figure S5b presents the absorption spectra of the corresponding neat films, where the onset wavelengths shift to 417 nm for **TPAP** and 513 nm for **TPAP1**. Compared to their solution spectra, both materials exhibit a redshift in λ_onset_—20 nm for **TPAP** and 30 nm for **TPAP1**. The more pronounced red-shift observed for **TPAP1** suggests stronger intermolecular interactions during film formation.

The passivation capabilities of the basic **TPAP** molecule and the functionalized **TPAP1** molecule have been analyzed by first-principles calculations.^38^ The freestanding **TPAP** and **TPAP1** molecules have been considered. Specifically, the distribution of partial charges has been determined based on the electrostatic potential (ESP) analysis; i.e., the partial atomic charges that fit the quantum mechanical electrostatic potential have been derived. In fact, the distribution of partial charges on the functional groups of the passivation molecules is paramount to understand the interaction at the interface between perovskite and passivation layer. In Figures 1b and 1c, the optimized geometry of the free-standing **TPAP** and **TPAP1** molecules is shown along with the related atomic partial charges. The basic **TPAP** passivator exhibits three pyridine functional groups where the nitrogen atom is negatively charged; thus, the **TPAP** is expected to passivate only one kind of defect, that is a positively charged defect, like under-coordinated lead at the perovskite surface. On the other hand, the functionalized **TPAP1** passivator presents, in addition to the two unmodified pyridine groups, a more intriguing terminal group composed of a negatively charged bromide ion and a positively charged pyridine cation, that is expected to interact with several kind of defects (e.g., both under-coordinated Pb^δ+^ and I^δ−^ on the perovskite surface), thus having improved passivation capabilities.

### 2.2 Interfacial Studies

The interaction between the passivation layer and the perovskite surface was first studied by simulation.^34,39^ In particular, we considered the adsorption characteristics of the **TPAP1** terminal group on the PbI_2_-terminated perovskite surface. The optimized configuration of this interacting system is represented in Figure 2a. As expected, the bromide ion interacts with the under-coordinated Pb^δ+^ on the perovskite surface, while the under-coordinated I^δ−^ interacts with the pyridine cation. With respect to the projected density of states (PDOS) of the perovskite slab reported in Figure 2a right panels (top), the passivation capability of **TPAP1** is demonstrated by the PDOS of the interacting system depicted in Figure 2a right panels (bottom), where the suppression of the perovskite surface states contributed by under-coordinated Pb^2+^ and I^–^ ions is pointed out by the red arrow. Note that the adsorption energy obtained for this configuration (i.e., *E*_ads_^**TPAP1**^ = −0.97 eV) is more favorable than the one related to the adsorption of the unmodified pyridine group (i.e., *E*_ads_^**TPAP**^ = −0.44 eV, as discussed in Figure S8), which confirms the suitability of **TPAP1** with respect to **TPAP**. The presence of iodine vacancies at the perovskite surface was then considered.^40^ As usual, we first obtained the optimized configuration for the interacting system (**TPAP1** terminal group + PbI_2_ terminated perovskite surface with V_I_ defects), which is shown in Figure 2b. Notably, we found that the bromide ion effectively replaces the missing iodine atom on the perovskite surface, interacting with both the Pb^δ+^ and MA^+^ cations, while the under-coordinated I^δ−^ again interacts with the pyridine cation. The presence of the defect states, highlighted in Figure 2b right panels (top), where the PDOS of the perovskite slab with V_I_ defects is represented, is effectively suppressed by the **TPAP1** passivator, as demonstrated by the PDOS of the interacting system reported in Figure 2b right panels (bottom). Note that, as expected, the adsorption energy obtained for this configuration (i.e., *E*_ads_^**TPAP1**^ = −2.03 eV) is much more favorable than the one derived for the unmodified pyridine group (i.e., *E*_ads_^**TPAP**^ = −0.56 eV, as discussed in Figure S9), confirming the **TPAP1** molecule as a more stable, effective, and suitable passivator with respect to the basic **TPAP** molecule.

**Figure 2:**
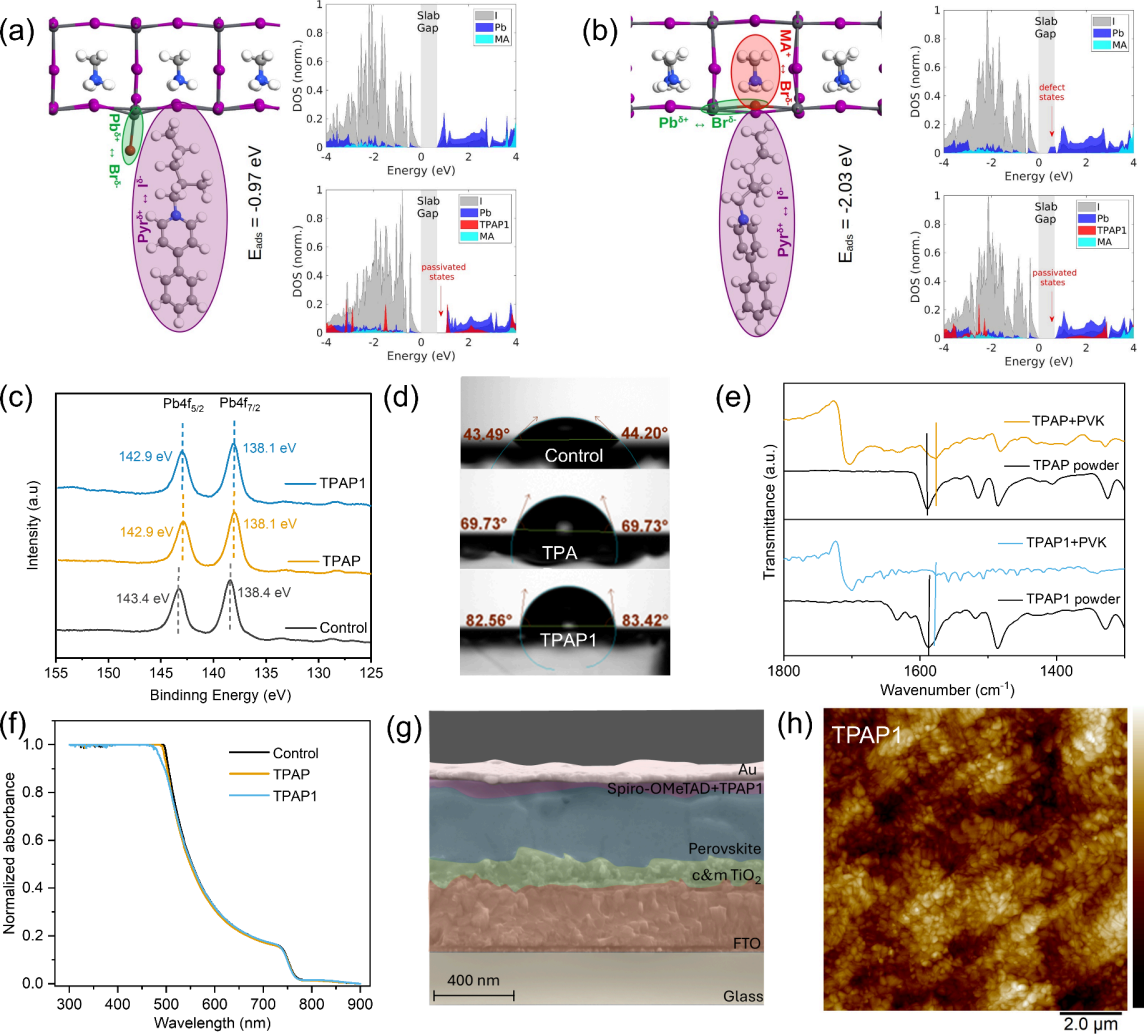
Optimized configuration for the functionalized terminal group of **TPAP1** interacting (a) with the PbI_2_ and (b) with the iodine vacancy (V_i_) terminated perovskite surface, where Pb, I, N, C, H, and Br atoms are represented in dark gray, purple, blue, gray, white, and red, respectively. The key interactions are highlighted by shaded areas, and the obtained adsorption energies are reported. The right panels show PDOS for the free-standing perovskite slab (top) and the interacting system (bottom), where the gap of the free-standing perovskite slab is reported for comparison. (c) High-resolution of Pb 4f component measured from passivator/perovskite and perovskite samples (**TPAP** and **TPAP1** were both applied at a concentration of 0.5 mM). (d) Contact angle measurement and (e) FT-IR and (f) UV–vis spectra comparing perovskite films before and after passivation. (g) Cross-section SEM and (h) AFM of perovskite film with **TPAP1**.

To further assess the interactions of the passivators with the perovskite layers, experiments were conducted for thin perovskite films with or without passivators. First, X-ray photoelectron spectroscopy (XPS) was employed to analyze potential variations in the core-level binding energy of lead, as shown in Figure 2c. The control sample exhibits two separate peaks at 138.4 and 143.4 eV, assigned to Pb 4f_7/2_ and Pb 4f_5/2_, respectively. Upon treatment with **TPAP** and **TPAP1**, these peaks exhibit downward shifts of 0.5 and 0.3 eV (now centered at 138.1 and 142.9 eV), indicating an increased electron density around Pb atoms. This shift suggests strong coordination between the pyridine groups of the passivators and under-coordinated Pb sites within the perovskite lattice.^41,42^ The contact angles of water droplets on these films were evaluated for assessing the surface wettability (Figure 2d). The contact angles on bare perovskite, perovskite/**TPAP**, and perovskite/**TPAP1** were measured as 44.2°, 69.73°, and 83.42°, respectively. The perovskite/**TPAP1** surface exhibited the largest water contact angle, which results from the hydrophobic characteristics of the 2-ethylhexyl group present in the ionic structure.^37^ This hydrophobicity indicates a surface that resists the adhesion of H_2_O and O_2_, making it more resistant to moisture, compared to other configurations. In addition, defects within the perovskite structure can serve as channels for moisture penetration, and the inhibition of defects by passivator may contribute to the improved stability of passivator-based PSCs. To better understand the interaction between the passivator and perovskite film, Fourier-transform infrared spectroscopy (FT-IR) was used to study the perovskite/passivator complexes. The FT-IR spectra (shown in Figure 2e) reveal a C–N stretching vibration at 1587 cm^–1^ in both **TPAP** and **TPAP1** powders, which shifts to 1576 cm^–1^ in the **TPAP**+PVK and **TPAP1**+PVK films. This observed change aligns with our expectations, and these peak shifts indicate a strong interaction between the passivators and the undercoordinated lead ions. X-ray diffraction (XRD) analysis was further employed to assess how the passivators affect the crystal structure of the perovskite film. As illustrated in Figure S10, the diffraction peak at 13.7° corresponds to the (100) crystal plane of the perovskite, while the characteristic peak at 12.4° is attributed to PbI_2_.^29^ The findings suggest that both molecules exert minimal influence on the perovskite crystal structure. Furthermore, the UV–vis of these samples suggests that the inclusion of small molecules does not alter the optical bandgap of the perovskite films (Figure 2f). The perovskite film’s surface features were investigated through scanning electron microscopy (SEM), as shown in Figure S11, whereas Figures 2g and S12, present cross-sectional images of a full device stack fabricated with **TPAP** and **TPAP1**. The perovskite solar cell samples, whether incorporating the passivation layer or not, displayed a similar morphology, indicating that the passivation layer has minimal impact on the perovskite morphology. We further investigated the morphology and contact potential difference (CPD) using atomic force microscopy (AFM) and Kelvin probe force microscopy (KPFM) measurements. Figure 2h and Figure S13 depict the surface morphology of perovskite films as well as those with **TPAP** and **TPAP1**. Measured root-mean-square (RMS) roughness values were 37.7 nm for the unmodified perovskite film, while **TPAP** and **TPAP1** passivation reduced the roughness to 14.1 and 23.9 nm, respectively. These results demonstrate that introducing passivation layers effectively reduces the surface roughness. Generally, a smoother interface is associated with fewer defects, which enhances charge carrier extraction at the interface.^30^ Thus, the smoother surface morphology induced by **TPAP** and **TPAP1** likely contributes to the observed improvement in the device performance. Additionally, an amorphous layer was observed on the **TPAP**-incorporated film surface, probably caused by a higher concentration of **TPAP**. The bare perovskite film had a CPD of 125.0 mV, corresponding to a work function of −5.12 eV (Figure S14). The calculated work function values for PVK/**TPAP** and PVK/**TPAP1** were −5.01 and −5.04 eV, respectively, suggesting that these passivators elevate the surface work function of the perovskite film, which should be favorable for charge transfer.^25^

### 2.3 Performance of Passivator-Based Solar Cells

Inspired by the promising features of the pyridine-based passivators, we constructed PSCs with an FTO/c-TiO_2_/m-TiO_2_/perovskite/Spiro-OMeTAD/Au structure (Figure 3a), incorporating **TPAP** or **TPAP1** via a spin-coating approach at the perovskite/Spiro-OMeTAD interfaces. The optimal concentrations of both passivating agents, yielding the best device performance, were found to be 15 mM in isopropanol (IPA) for **TPAP** and 0.5 mM in IPA for **TPAP1** (Figures S15 and S16). Notably, the ionic passivator (**TPAP1**) requires an order of magnitude lower concentration than the nonionic passivator (**TPAP**) for effective passivation. The PCE­(i) and other performance metrics obtained from the 20 best-performing devices measured in the air for each passivator are displayed in Figure 3b, Figure S17, and Table 2. Compared to the control device (29.2%), the PCE­(i) increased to 30.1% for **TPAP**-modified devices and 31.7% for **TPAP1**-modified devices. The performance enhancement was mainly due to improvements in the *V*_OC_ and FF. Under 1000 lux (∼0.32 mW cm^–2^), *V*_OC_ rose from 0.95 to 1.00 V, while FF elevated from 76.6% to 79.3% (Figure S17). Remarkably, the *V*_OC_ achieved by **TPAP1**-modified devices reached 1.00 V, which is comparable to the highest reported value of 1.08 V for perovskites with a ∼1.60 eV bandgap under low-light conditions (LED 3376 K, 984 lux), as summarized in Table S1 and Figure S18. A comparative analysis was also performed using phenylethylammonium iodide (PEAI), a common reference passivator, under identical fabrication conditions. The results showed that **TPAP1** demonstrates superior photovoltaic performance relative to PEAI, while **TPAP** achieves similar efficiency, confirming the strong passivation properties of both new molecules (Figure S19). Additionally, both the control and modified devices were evaluated under 1 sun (100 mW cm^–2^) illumination. The control device showed a power PCE of 18.3%, whereas the modified device demonstrated an improved efficiency of 18.8% (Figure S20). The improvements in both *V*_OC_ and FF indicate a reduction in the interfacial defect density and an increase in the charge transfer efficiency, indicating improved interfacial quality and carrier dynamics. Notably, the performance enhancements were more pronounced under indoor lighting conditions, underscoring the effectiveness of **TPAP** and **TPAP1** in low-light environments. This performance enhancement is further illustrated in Figure 3c, which displays the current density–voltage (*J–V*) curves. To verify the accuracy of the PCE results, stable power output (SPO) was conducted under maximum power point tracking (MPPT, Figure S21). The obtained current density values exhibited less than a 4% deviation from the integrated current density extracted from external quantum efficiency (EQE) measurements (Figure 3d), confirming the consistency of the data. Overall, the efficiency improvements observed in these devices result from the effective chemical passivation of **TPAP** and **TPAP1**.

**Figure 3:**
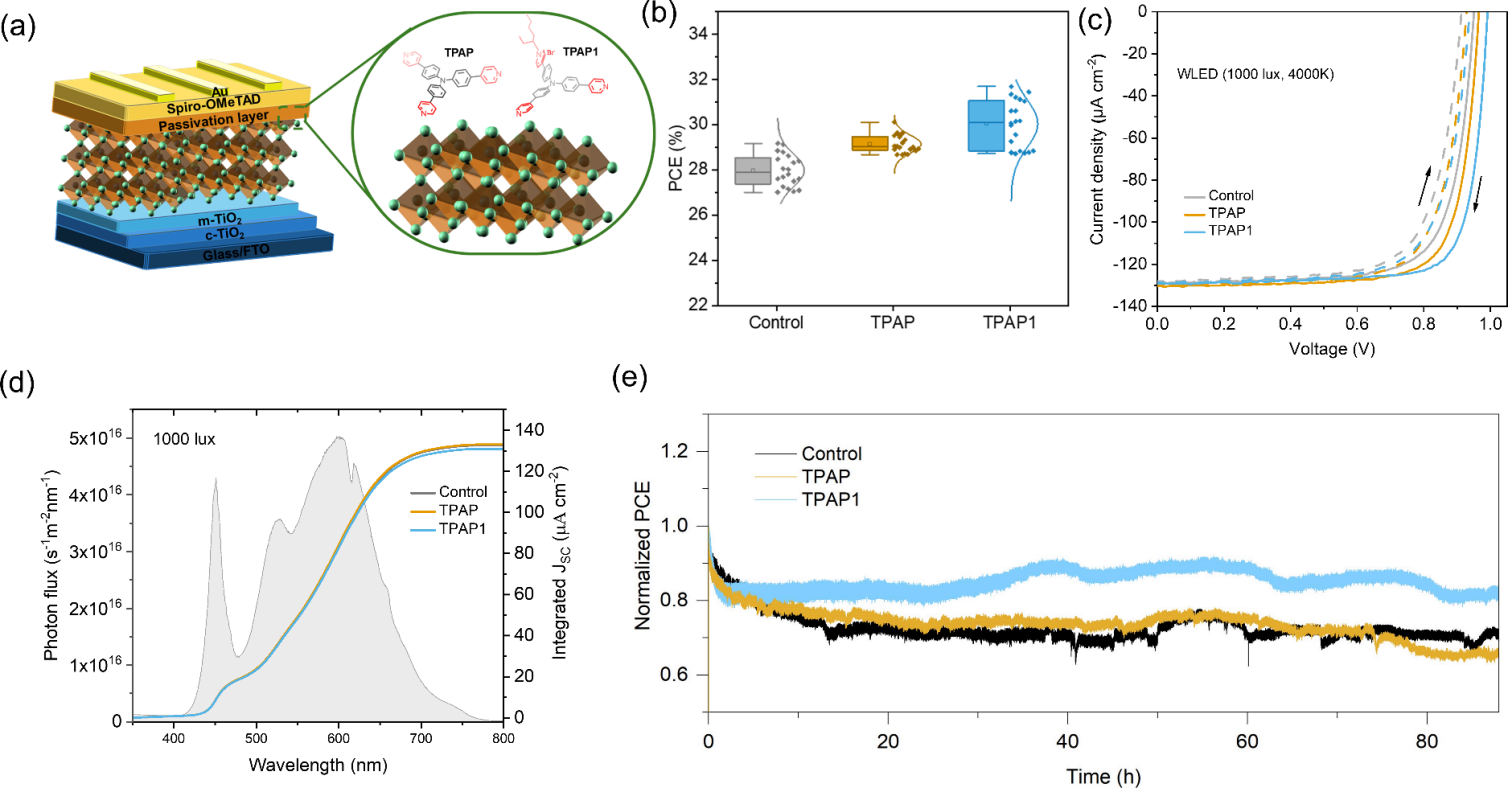
(a) Schematic diagram of the device architecture used in this work and the interaction between **TPAP** and **TPAP1** with defects on the perovskite surface. (b) The statistical distribution of PCE from 20 devices for each condition under 1000 lux WLED (4000 K) illumination. Box-and-whisker plots illustrate the statistical distribution of the data. The line inside each box indicates the median; the box boundaries represent the standard deviation; and the whiskers denote the outliers. (c) *J–V* curves for PSCs with and without passivators at 1000 lux. (d) Photon flux intensity spectrum of the 1000 lux WLED and the corresponding integrated JSC curves for the best-performing PSCs. (e) Normalized long-term stability at MPP tracking under continuous 1000 lux WLED (4000 K) illumination in N_2_ atmosphere at room temperature for PSCs with and without passivators.

**Table 2:** Averaged Photovoltaic Characteristics of 20 Solar Cells of Passivator-PSCs under WLED (1000 lux, 4000 K)^a^

passivator	PCE(i) [%]	*V*_OC_ [V]	*J*_SC_ [mA cm^–2^]	FF [%]
control	28.0 ± 1.2 (29.2)	0.93 ± 0.02 (0.95)	0.126 ± 0.003 (0.123)	75.8 ± 3.0 (76.6)
**TPAP**	29.1 ± 1.0 (30.1)	0.94 ± 0.02 (0.97)	0.126 ± 0.004 (0.130)	76.8 ± 2.7 (76.9)
**TPAP1**	30.0 ± 1.7 (31.7)	0.97 ± 0.02 (1.00)	0.125 ± 0.004 (0.129)	77.2 ± 3.4 (79.3)

a Bracketed values represent the data from the top-performing device.

In addition to performance evaluation, understanding the effect of molecular introduction on the long-term MPPT is essential. To investigate this, we performed MPPT according to the ISOS-L-1I protocol.^43^ The devices were exposed to continuous 1000 lux WLED (4000 K) illumination under N_2_ atmosphere to evaluate long-term stability (Figure 3e). The results indicate that the devices modified with **TPAP1** retain 83% of their initial PCEs after 90 h, whereas the devices modified with **TPAP** and control devices retain only 70% and 74% of their initial photovoltaic performance, respectively. The improved stability is attributed to the hydrophobicity of the **TPAP1** films and the decrease in defect density. Furthermore, to evaluate the moisture stability of the passivated devices, we monitored changes in PCE over time under ambient conditions (RH ≈ 47%, *T* ≈ 22 °C) using periodic *J*–*V* measurements. The results, presented in Figure S22, demonstrate that devices treated with **TPAP** and **TPAP1** retain their performance more effectively than the control group, emphasizing the enhanced protection afforded by the passivation layers against humidity-induced degradation.

### 2.4 Defect Physics of Passivator-Based Solar Cells

To examine the influence of pyridine-based passivation regarding charge carrier recombination, steady-state (PL) and time-resolved photoluminescence (TRPL) measurements were conducted by using perovskite films coated on glass substrates. The PL results showed a significant enhancement in the perovskite films modified with passivators compared to the control samples (Figure 4a), suggesting effective suppression of surface trap-assisted recombination.^44^ Additionally, as illustrated in Figure 4b and Table S2, the decay from TRPL results of perovskite films modified with **TPAP** and **TPAP1** was significantly slower than those of the control sample, consistent with the PL results. The decay time of the perovskite films modified by **TPAP** and **TPAP1** showed significant enhancements compared to the control sample (522 ns), with values of 749.4 and 1059 ns, respectively. These results indicate that **TPAP** and **TPAP1** provide superior chemical passivation compared to control cells,^25,44^ which is consistent with the findings. From these results, we can infer that the introduced molecule provides advantages in chemical passivation at the perovskite/HTL interface, thereby enhancing the performance of the perovskite devices.

**Figure 4:**
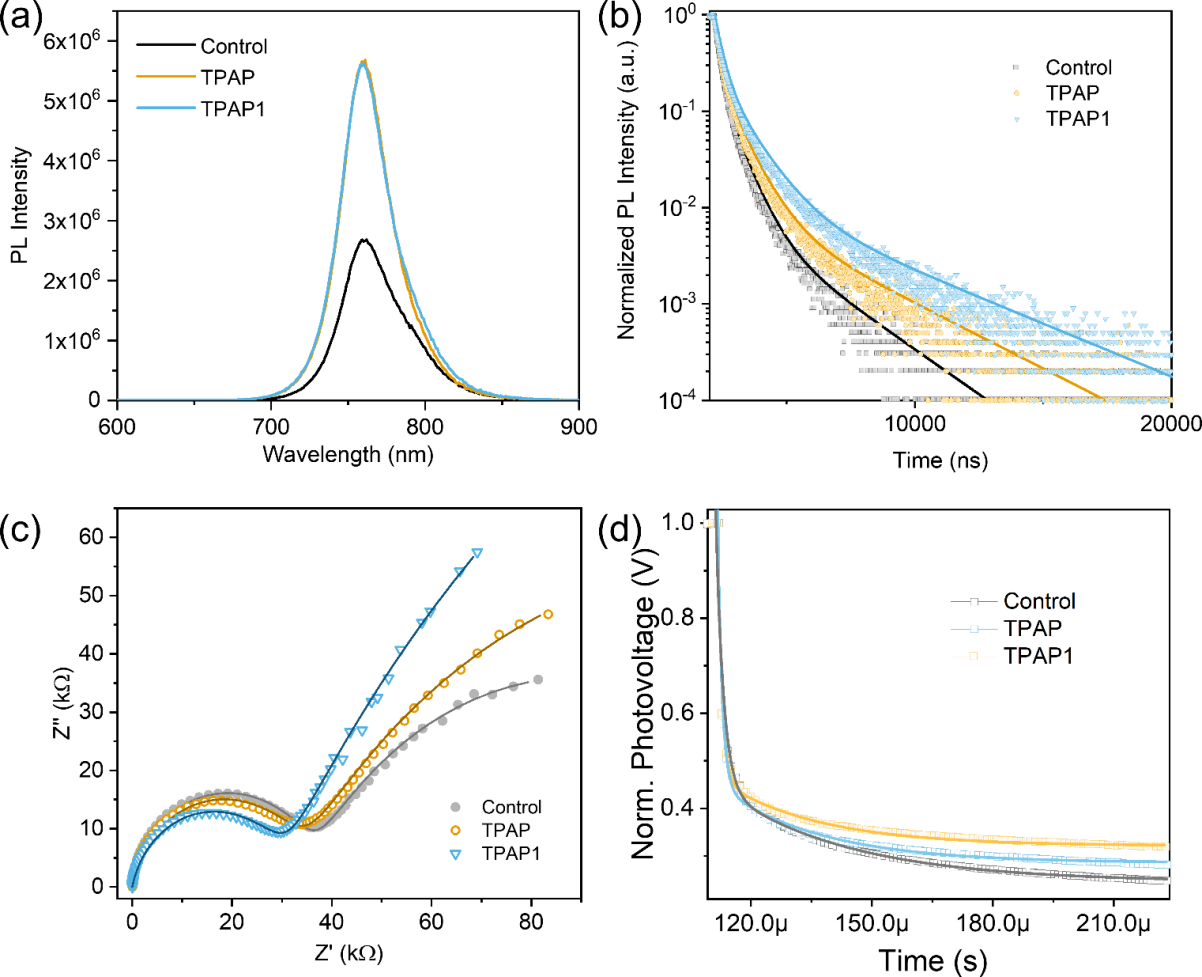
(a) PL and (b) TRPL spectra of perovskite films deposited on glass substrates excited by a 405 nm laser. (c) Nyquist plots of the devices; symbols indicate experimental data, and solid lines represent fits based on the equivalent circuit model. (d) TPV decays of perovskite devices with or without passivators under a high-frequency light source.

The *J–V* curves of PSCs treated with **TPAP** and **TPAP1** were taken under varying light intensities (*P*_light_), and the related *J*_SC_–*P*_light_ and *V*_OC_–*P*_light_ curves are illustrated in Figure S23.^45^ Based on the formula α = ln­(*J*_SC_)/ln­(*P*_light_), the slope (α) values for the control, **TPAP**, and **TPAP1** devices were determined to be 1.16, 1.03, and 1.04, respectively. The α values approaching unity for the passivator-treated devices suggest suppressed monomolecular recombination within the device structure. The α values nearing 1 for the devices incorporating passivators imply a reduction in monomolecular recombination processes within the device architecture.^46^ The *V*_OC_–*P*_light_ curves were fitted using the equation *V*_OC_ = (*nkT*/*q*) ln­(*P*_light_), where *k* represents the Boltzmann constant, *T* is the absolute temperature, and *q* denotes the elementary charge, allowing extraction of the ideality factor *n*. The *n* represents the diode ideality factor, a key parameter that characterizes the primary recombination mechanisms and the splitting of the Fermi level.^47^ For a classical p–n junction, the ideality factor usually falls between 1 and 2. A value of 1 corresponds to recombination dominated by bimolecular processes, whereas an ideality factor near 2 indicates that the recombination is primarily nonradiative, often due to Shockley–Read–Hall (SRH) recombination. Although in perovskite optoelectronics, the ideality factor (*n*_id_) often exceeds 2 due to the strong coupling between electronic and ionic charge carriers at the cathode interface. The obtained *n* values were utilized to evaluate the extent of trap-assisted recombination,^28^ with larger *n* values indicating a higher degree of such recombination. The extracted ideality factors (*n*_id_) for the control-, **TPAP**-, and **TPAP1**-based PSCs are 2.56, 2.51, and 2.43, respectively. These results indicate that the incorporation of passivating agents effectively reduces trap-assisted recombination, aligning well with the results of trap density measurements. Likewise, the dark current of the PSCs with or without passivators was recorded to extract current leakage and shunt resistance (*R*_sh_), reflecting the extent of charge trapping within the device (Figure S24).^48^ The device without a passivation layer exhibits a huge leakage current density of 2.57 × 10^–3^ mA·cm^–2^ and a relatively low *R*_sh_ value 1.38 × 10^7^ Ω·cm^2^. Nevertheless, after inserting a passivation layer onto the perovskite, the dark current density is significantly suppressed to 1.11 × 10^–3^ mA·cm^–2^ for **TPAP** and further to 6.29 × 10^–4^ mA·cm^–2^ for **TPAP1**. Concomitantly, the *R*_sh_ values increase to 1.55 × 10^7^ and 2.91 × 10^7^ Ω·cm^2^, respectively, demonstrating the crucial role of the passivation layer in preventing leakage. The reduced leakage current observed in passivated devices confirms a decrease in charge trap densities at the perovskite–passivator interface, leading to suppressed nonradiative recombination (Figure S25). As a result, the passivated devices exhibit higher *R*_sh_, lower dark currents, and reduced *n*_id_, which reflect minimized defect density and are key factors contributing to improved *V*_OC_ and overall PCE of the device.

Electrochemical impedance spectroscopy (EIS) was employed to analyze the charge transport dynamics and internal resistance within the perovskite/HTL interfaces, which support the observed enhancements in PCE­(i). The Nyquist plots of the devices are depicted in Figure 4c, and the corresponding equivalent circuit model employed for fitting the experimental data is provided in Figure S26. The impedance spectrum exhibits a small arc in the high-frequency region and a pronounced semicircular feature at low frequencies. The applied circuit model comprises two *R*(CPE) components, where *R* denotes resistance and CPE represents a constant phase element, connected in series. Within this model, *R*_S_ accounts for the series resistance, while *R*_ct_ and CPE1 correspond to the charge transfer resistance and the interfacial capacitance at high frequencies, respectively, typically arising from nonidealities at the interfaces.^49,50^ Conversely, *R*_rec_ and CPE2 are attributed to recombination processes and capacitive behavior within the bulk perovskite and passivation regions at low frequencies. Since *R*_ct_ in the high-frequency region is associated with charge carrier extraction, the reduced *R*_ct_ values of 3.23 and 2.9 kΩ for the **TPAP** and **TPAP1** devices, compared to 3.51 kΩ for the control device, indicate improved charge extraction. This correlates well with the enhanced FF observed in the devices incorporating passivators. The low-frequency semicircle, governed by the recombination resistance (*R*_rec_), reveals elevated values for **TPAP** (18.6 kΩ) and **TPAP1** (40.4 Ω) relative to the control (11 kΩ), suggesting more effective inhibition of charge recombination.^51^ The EIS data, showing reduced *R*_ct_ values and enhanced *R*_rec_ values with the **TPAP** and **TPAP1** passivators, suggest that the passivators effectively suppress trap states and nonradiative recombination, supporting the PL and TRPL results. Transient photovoltage (TPV) measurements were conducted on all three devices to further understand their recombination kinetics.^52,53^ The TPV decay spectra were analyzed using a biexponential decay function, which reveals the presence of two distinct recombination processes or pathways affecting carrier dynamics. As calculated from Figure 4d, Figure S27, and Table S3, the average lifetimes for the control, **TPAP**, and **TPAP1** devices were determined to be 24.9, 4.91, and 3.72 μs, respectively. These results suggest that recombination is increasingly suppressed in the **TPAP** and **TPAP1** devices compared to in the control device. Furthermore, the **TPAP1** device exhibits the longest average lifetime, indicating minimal Shockley–Read–Hall (SRH) recombination, which aligns with its superior device performance and the trends observed in the EIS measurements.

## 3 Conclusions

In this work, two pyridine-based compounds, **TPAP** and **TPAP1**, were designed to improve the efficiency and stability of PSCs by providing effective surface passivation. The pyridine moiety in these passivators chemically interacts with under-coordinated lead ions at the surface of the perovskite, effectively lowering the defect density. Notably, the ionic compound **TPAP1** achieves multiple passivation effects, enabling better passivation at a lower concentration of 0.5 mM. This is due to the pyridinium cation being able to passivate negatively and positively charged sites, while the 2-ethylhexyl bromide organic group provides higher hydrophobicity, leading to improved surface stability. These synergistic effects promote enhanced hole extraction and charge transfer efficiency at the perovskite/Spiro-OMeTAD interface by suppressing recombination and inhibiting charge accumulation, leading to enhanced *V*_OC_ and FF. As a result, PSCs incorporating **TPAP** and **TPAP1** achieved remarkable PCEs­(i) of 30.1% and 31.7%, respectively, along with improved device stability. The enhanced passivation not only contributes to a higher device efficiency but also mitigates environmental degradation, extending operational stability. This study demonstrates an effective and scalable passivation strategy for advancing perovskite photovoltaics, emphasizing the critical role of multifunctional organic passivators in improving surface passivation and the overall device performance.

## 4 Experimental Section

### 4.1 Materials

Thirty NRD titanium dioxide (TiO_2_) paste, methylammonium bromide (MABr), and formamidinium iodide (FAI) were obtained from Greatcell Solar Materials. Lead iodide (PbI_2_, >98%) and lead bromide (PbBr_2_, >98%) were obtained from TCl. Cesium iodide (CsI) was aquired from abcr. Stock solution (TDBA) 75 wt % in isopropanol of titanium diisopropoxide bis­(acetylacetonate), DMSO (dimethylsulfoxide, anhydrous, ≥99.9%), DMF (dimethyl formamide, anhydrous, 99.8%), CB (chlorobenzene, extra dry, 99.8%), bis­(trifluoromethane)­sulfonimide lithium salt (Li-TFSI, 99.95%), 4-tert-butyilpyridine (4-tBP, 98%), and acetonitrile (99.9%) were acquired from Sigma-Aldrich. FK209 Co­(III) (tris­[2-(1H-pyrazol-1-yl)-4-tert-butylpyridine]­cobalt­(III)­tri­[bis­(trifluoromethane) sulfonimide], >98%) was purchased from Dyenamo. Spiro-OMeTAD (2,2′,7,7′-tetrakis (*N*,*N*-di-*p*-methoxy phenylamino)-9,9-spirobifluorene) was acquired from Lumtec. FTO coated glass substrates of 2 cm × 2 cm were purchased from Yingkou Opv Tech New Energy Technology Co.

### 4.2 Characterization

#### 4.2.1 Characterization Using Spectroscopic and Thermal Methods

UV–vis absorption spectra were collected both in solutions (*o*-dichlorobenzene ≈ 10^–5^ M) and deposited as films on a Shimadzu UV-1800 absorption spectrometer (dual-beam grating), Shimadzu Corporation, Japan. Steady-state photoluminescence (PL) spectra of the films were collected by using an FLS1000 spectrofluorometer from Edinburgh Instruments (UK). Time-resolved photoluminescence (TRPL) decay measurements were carried out with a FluoTime 300 high-end photoluminescence spectrometer through time-correlated single-photon counting (TCSPC) techniques. Detection was performed by using a Hamamatsu R3809U-50 microchannel plate photomultiplier in a 90° configuration. For thermogravimetric analysis (TGA), a Netzsch TG 209 F3 instrument was employed operating under a nitrogen atmosphere (40 mL/min) with a heating rate of 10 K/min. The FT-IR spectra of the passivators were recorded using the INVENIO FT-IR Spectrometer Platform alongside passivators/perovskite complexes prepared by mixing equimolar amounts of the passivators and the perovskite in a DMSO/DMF solution and evaporating at 100 °C.

#### 4.2.2 Electrochemical Characterization

The cyclic voltammetry experiments was performed using an Ivium Technologies CompactStat.h instrument in THF, operating at a scan rate of 100 mV/s. Potentials were referenced against a Ag/Ag^+^ quasi-reference electrode, aligned using ferrocene (Fc) before each experiment. To determine the HOMO energy level of the HTMs, measurements were taken from the onset of the half-wave potential during the oxidation process. The HOMO and LUMO energies were then derived using eqs 1 and 2, as outlined below:

$$E_{\mathrm{HOMO}}=-\left[E_{\mathrm{ox}}-E_{\left(Fc/{Fc}^{+}\right)}+5.10\right]\;\left(\mathrm{eV}\right)$$ 1

$$E_{\mathrm{LUMO}}=E_{\mathrm{HOMO}}+E_{g}\;\left(\mathrm{eV}\right)$$ 2

The value *E*_(Fc/Fc^+^)_ refers to the half-wave potential of the Fc/Fc^+^ couple, while *E*_ox_ is the half-wave potential of the HTM/HTM^+^ couple. The optical band gap, *E*_g_, is calculated using *E*_g_ = 1240/λ_onset_ (eV) (where λ_onset_ is the onset wavelength of the passivator’s absorption measured in solution).

#### 4.2.3 Computational Details

All the simulations have been performed using the QuantumATK software package,^38^ with the GGA-PBE pseudopotentials and the LCAO basis set available therein. The default settings for the accuracy of the calculations have been employed, in particular, k-point density of 4 × 4 × 4 Å^3^ and force convergence threshold for geometry optimization of 0.05 eV/Å. The perovskite surface has been modeled as a 2 × 2 × 2 MAPbI_3_ slab in its pseudocubic phase, exposing its PbI_2_ surface. For the VI surface defects, only one iodine over the eight total surface iodine atoms is missing.

The water contact angle (CA) measurements for perovskite films, which were spin-coated onto FTO substrates prepared according to the full solar cell fabrication process, were performed by using an Attension Theta Lite optical goniometer from Biolin Scientific AB (Sweden). Similar samples were also performed with a Bruker Dimension Icon atomic force microscope (AFM) to measure surface roughness as well as work function via Kelvin probe force microscopy (KPFM). Bruker’s SCM-PIT-V2 probe was used for these measurements, and they were conducted in ambient air.

X-ray photoelectron spectroscopy (XPS) analyses were carried out under ultrahigh vacuum conditions using an ESCA Model 3000 instrument from Omicron Nanotechnology GmbH. During these measurements, the base pressure was maintained below 1 × 10^–10^ mbar. A focused monochromatized Al Kα radiation (*h*ν = 1486.5 eV) was employed as an excitation source for X-ray photoelectron spectroscopy. Data acquisition, processing, and analysis were carried out using CasaXPS version 2.3.22 PR1.0. After Shirley background subtraction was applied, peak analysis was performed using an asymmetric Gaussian–Lorentzian function to best represent the line shapes of the spectral components. The binding energy scale was standardized by referencing the C 1s (C–C) peak to 284.8 eV.

#### 4.2.4 X-ray Diffraction (XRD) Analysis

XRD patterns were obtained by using a Malvern Panalytical Empyrean diffractometer equipped with a Cu Kα X-ray source (λ = 0.15418 nm). The instrument was utilized at 45 kV and 40 mA. The films were scanned over 10–60° with a 0.026° step size and step duration of 2998 s/step. The crystal structures were illustrated using the VESTA visualization program. Structural details were obtained through Rietveld refinement with the GSAS software suite. The phase purity of the synthesized samples was determined by performing Rietveld refinement on the XRD data, considering comprehensive adjustments of both crystallographic and instrumental parameters using the GSAS software suite.

#### 4.2.5 Scanning Electron Microscopy (SEM)

Both top-view and cross-sectional images of the films and devices were obtained using a Zeiss UltraPlus field emission scanning electron microscope (FE-SEM) operating at 3 kV. In addition, elemental analysis was conducted via EDS pectroscopy with an Oxford Instruments X-MaxN 80 EDS detector paired to the Zeiss UltraPlus FE-SEM, allowing for precise determination of the film’s composition.

#### 4.2.6 Transient Photovoltage Measurements (TPV)

TPV was performed using a homemade setup with a white light source with 1 sun intensity.

A biexponential decay function can typically be described as

3 $$\Delta V\left(t\right)=A_{1}\exp\left(-\frac{t}{\tau_{1}}\right)+A_{2}\exp\left(-\frac{t}{\tau_{2}}\right)$$

where Δ*V* is photovoltage, *A*_1_ and *A*_2_ are the amplitudes associated with each recombination process, and τ_1_ and τ_2_ are two distinct lifetimes (or time constants) associated with the fast and slow recombination processes, respectively.

### 4.3 Fabrication

#### 4.3.1 Solar Cell Fabrication

The photovoltaic devices were prepared using glass substrates coated with fluorine-doped tin oxide (FTO), which were sourced from OPV Tech. Each substrate featured dimensions of 2.54 × 2.54 cm and a thickness of 0.22 cm. To maximize cleanliness, the substrates were first brushed with a 2% Mucasol solution prepared in deionized water. This was followed by sequential ultrasonic cleaning in deionized water, acetone, and 2-propanol, each for 15 min. After drying with a stream of nitrogen, the substrates underwent a 10 min UV-ozone treatment to prepare them for subsequent fabrication steps.

A compact TiO_2_ (c-TiO_2_) layer was applied to the substrates using spray pyrolysis at 450 °C, followed by a 45 min sintering process at the same temperature in air. The coating solution was prepared by diluting a titanium diisopropoxide bis­(acetylacetonate) stock solution in 2-propanol, using 1.15 mL of stock per 5 mL of solvent. Next, a mesoporous TiO_2_ (m-TiO_2_) layer was formed by spin-coating 80 μL of 30 NRD TiO_2_ nanoparticle paste (150 mg/mL in ethanol) at 4000 rpm for 10 s, with an acceleration rate of 2000 rpm/s. This layer was briefly annealed at 100 °C, then calcined at 450 °C for 30 min, with a ramp-up time of 45 min. Afterward, the substrates were cooled to 200 °C and transferred to a nitrogen-filled glovebox for the remaining fabrication steps.

The triple-cation perovskite CsMAFA-Pb (with the formula (Cs_*x*_(MA_0.17_FA_0.83_)_(100–*x*)_Pb­(I_0.83_Br_0.17_)_3_) was synthesized following the method explained by Saliba et al. To prepare the perovskite precursor solution, FAI (0.95 M), MABr (0.19 M), PbBr_2_ (0.20 M), and PbI_2_ (1.1 M) were mixed in a solvent blend of DMSO and DMF in a 1:4 volume ratio, 1 day before film deposition. A 1.5 M solution of CsI was prepared in DMSO, after which 40 μL of this stock was introduced into 1 mL of the perovskite precursor. The resulting mixture was stirred thoroughly for approximately 24 h before being used in subsequent steps.

For film fabrication, 60 μL of the perovskite precursor solution was dispensed onto the FTO|c-TiO_2_|m-TiO_2_ substrate and spin-coated at 1000 rpm for 10 s, followed by 6000 rpm for 20 s. Five seconds before the end of spin coating, 100 μL of chlorobenzene was dropped onto the spinning substrate as an antisolvent. After preparation, the films underwent an annealing process at 110 °C for one hour. When incorporating passivators, 100 μL of a passivator solution in IPA was dynamically spin-coated onto the rotating substrate at 4000 rpm for 30 s. After deposition of the passivation layer, Spiro-OMeTAD was subsequently deposited as the hole transport layer (HTL). The Spiro-OMeTAD solution was made by dissolving Spiro-OMeTAD in chlorobenzene at a concentration of 36.2 mg/mL. For the doped formulation, 14.4 μL of tBP, 8.8 μL of Li-TFSI (at a concentration of 520 mg/mL in acetonitrile), and 14.5 μL of FK209 Co­(III) (300 mg/mL in acetonitrile) were sequentially added to 1 mL of the Spiro-OMeTAD solution, ensuring thorough mixing. An 80 μL sample of this mixture was then spin-coated onto the perovskite films at 1800 rpm for 30 s. Afterward, the samples were stored in a dry cabinet maintained at roughly 15–20% relative humidity overnight before depositing the top electrode. Finally, an 80 to 100 nm thick layer of gold was thermally evaporated onto the samples using the OPTIvap Vacuum Deposition Tool (MBraun), completing the photovoltaic devices with an active area of 7 mm^2^.

#### 4.3.2 Device Characterization

To assess device performance, a Litos Lite system from FLUXiM AG (Switzerland) was employed to record both reverse and forward *J*–*V* sweeps at a scan rate of 50 mV/s, as well as maximum power point (MPP) tracking for the fabricated photovoltaic cells under simulated sunlight. *J*–*V* curves and stable power output (SPO) measurements were conducted in air, while extended MPP tracking was performed in a nitrogen atmosphere. All measurements utilized 7 mm^2^ aperture masks.

A Philips HUE WLED bulb, set to a color temperature of 4000 K, served as the light source for indoor photovoltaic measurements. Illumination was monitored using a calibrated Digi-Sense lux meter, while power density at various light levels was determined with a Konica Minolta CL-500A Illuminance Spectrophotometer. For instance, at an intensity of 1000 lux, the WLED bulb delivered approximately 0.32 mW cm^–2^ to the cell surface. Both forward and reverse *J*–*V* sweeps at a scan rate of 50 mV/s, as well as stable power output (SPO) measurements, were recorded using a Keithley 4250 source-monitor unit with a 4-wire configuration. All measurements were conducted within a black box, utilizing 10 mm^2^ aperture masks for precise evaluation. Maximum power point (MPP) tracking under indoor lighting was handled with a Keithley 4250 source-monitor unit in a four-wire configuration operating in a nitrogen atmosphere under continuous illumination. For indoor MPP tracking, a Philips HUE WLED bulb was set to deliver 1000 lux (approximately 0.32 mW/cm^2^). External quantum efficiency (EQE) spectra were obtained across the 350 to 850 nm range using a QuantX-300 (Newport) system.

### 4.4 Synthetic Procedures

#### 4.4.1 Synthesis of Tris­(4-(pyridin-4-yl)­phenyl)­amine (**TPAP**)

Tri­(4-bromophenyl)­amine^54^ (1.00 g, 2.10 mmol), pyridine-4-boronic acid (1.15 g, 9.35 mmol), Pd­(PPh_3_)_4_ (0.353 g, 0.3 mmol), and K_2_CO_3_ (0.552 g, 4.00 mmol) were introduced into a mixture of 1,4-dioxane (50 mL) and water (10 mL). The resulting solution was degassed via three argon/vacuum cycles and subsequently refluxed under argon atmosphere for 24 h. After completion, the solvent was evaporated under a vacuum, and the resulting bright yellow solid was then dissolved in dichloromethane. The organic phase was washed three times with water, dried with anhydrous MgSO_4_, and the solvent was removed to afford a yellow crude product. The target compound was obtained as a yellow solid after silica gel column chromatography. Yield: 0.67 g (68%). ^1^H NMR (500 MHz, CDCl_3_): 8.64 (d, 6H), 7.63 (m, 6H), 7.54 (m, 6H), 7.29 (m, 6H).

#### 4.4.2 Synthesis of 4-(4-(Bis­(4-(pyridin-4-yl)­phenyl)­amino)­phenyl)-1-(2-ethylhexyl)­pyridin-1-ium (**TPAP1**)^37^

**TPAP** (100 mg, 0.21 mmol) was dissolved in 25 mL of anhydrous DMF/DMSO (3:1, v/v) in a 50 mL round-bottomed flask under nitrogen gas. The temperature of the solution increased to 100 °C with continuous stirring, with 2-ethylhexyl bromide (1.05 equiv) added gradually dropwise. The reaction mixture was refluxed for 48 h under continuous monitoring by TLC. Once the reaction was complete, the solvent was evaporated under vacuum with a rotary evaporator. The crude product was then purified by multiple washes with dichloromethane to give **TPAP1** as an orange solid (84 mg, 68% yield). Owing to their poor solubilities, the ^13^C NMR of **TPAP1** cannot be measured in Methanol-D_4_ solution. ^1^H NMR (500 MHz, Methanol-D_4_): 8.91 (d, 2H), 8.82 (d, 4H), 8.40 (m, 6H), 8.08 (m, 6H), 7.41 (m, 6H), 4.52 (d, 2H), 2.16 (s, 1H), 1.45–1.22 (m, 8H), 0.96 (t, 3H), 0.90 (t, 3H). HRMS (*m*/*z*, ES+) calcd for C_41_H_41_N_4_ 589.3325, found 589.3322.

## Supplementary Material



## Notes

NMR and mass spectra; thermal analysis; UV–vis and PL spectra; cyclic voltammetry; DFT calculations; XRD and microscopy images; device performance data under indoor and 1 sun illumination; TRPL, *J*–*V*, EIS, and TPV analysis (PDF)
