# Supplementary material for: Pyridine-Based Multifunctional Surface Passivators Enable Efficient and Stable Perovskite Indoor Photovoltaics
Source: ACS Appl Mater Interfaces. 2025 Aug 21;17(35):49409–20. doi: 10.1021/acsami.5c08539 (PMC12412104; doi:10.1021/acsami.5c08539)
Supplement: Supplementary file 1 [file am5c08539_si_001.pdf]

Supporting Information

Pyridine-based Multifunctional Surface Passivators Enable Efficient and Stable Perovskite Indoor Photovoltaics

Yi Han[a+], Ceylan Doyranli[a+], Alessia Di Vito[b], Matthias Auf der Maur[b], Mokurala Krishnaiah [a], Paavo Mäkinen[a], Ramesh Kumar[c], Basheer Al-Anesi[a], Debjit Manna[a], Paola Vivo[a]*

[a] *Hybrid Solar Cells, Faculty of Engineering and Natural Sciences, Tampere University, Tampere FI-33014, Finland*

[b] *Department of Electronic Engineering, University of Tor Vergata, Rome, 00133, Italy*

[c] *Department of Chemistry – Ångström Laboratory, Uppsala University, Uppsala SE-75120, Sweden*

* Email: paola.vivo@tuni.fi

[+]*Y.H. and C.D. contributed equally to this work*

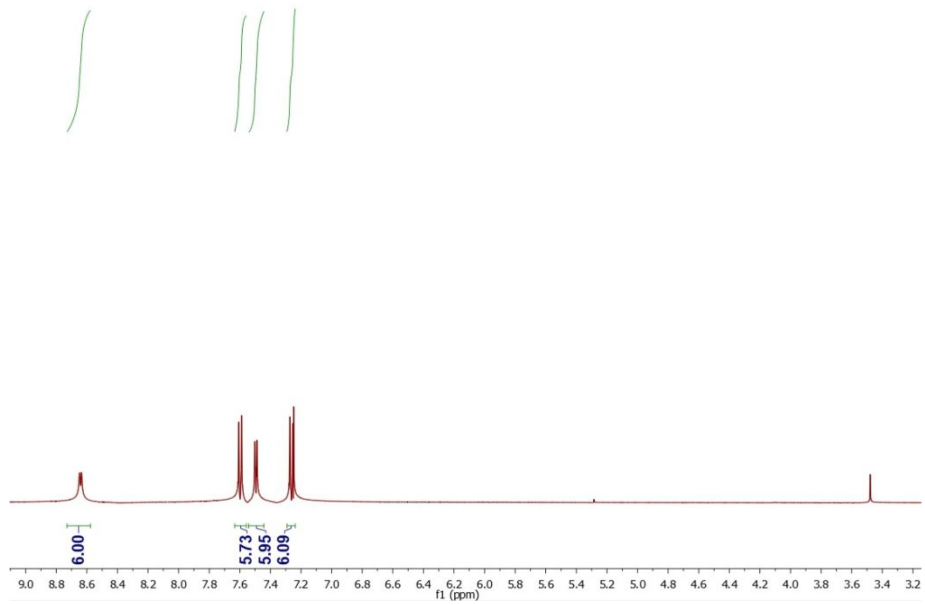

Figure S1. ^{1}H NMR spectrum of **TPAP** in $CDCl_3$.

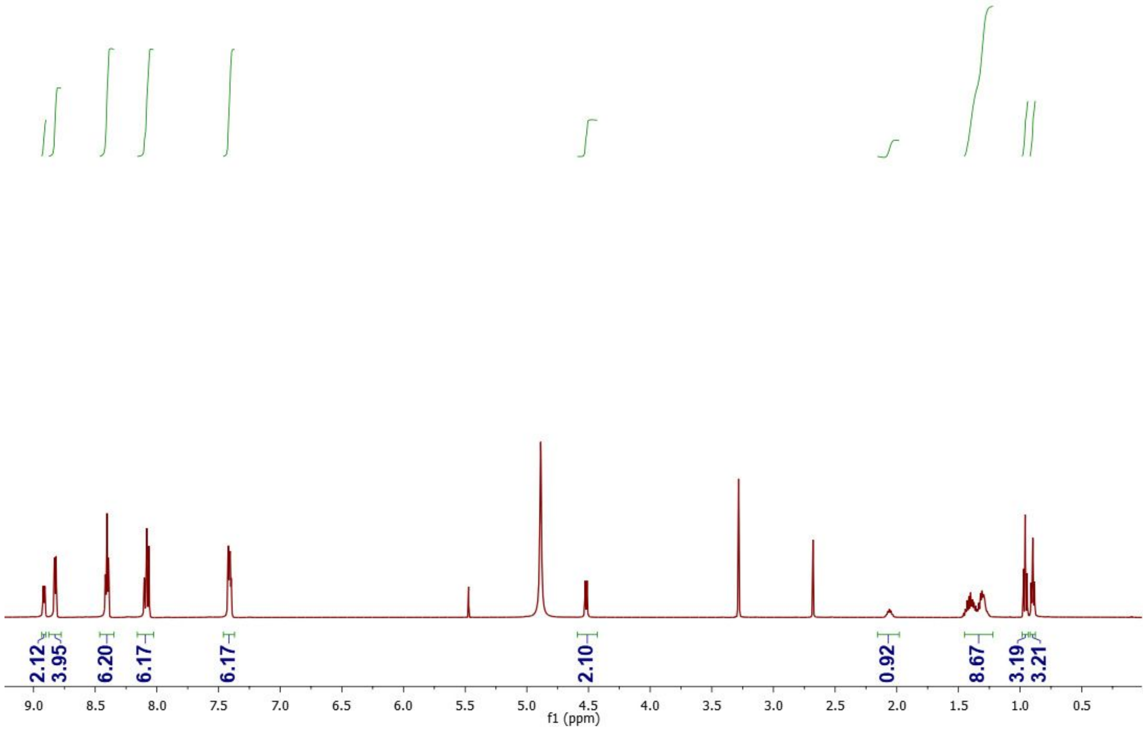

Figure S2. ^{1}H NMR spectrum of **TPAP1** in Methanol-D_4.

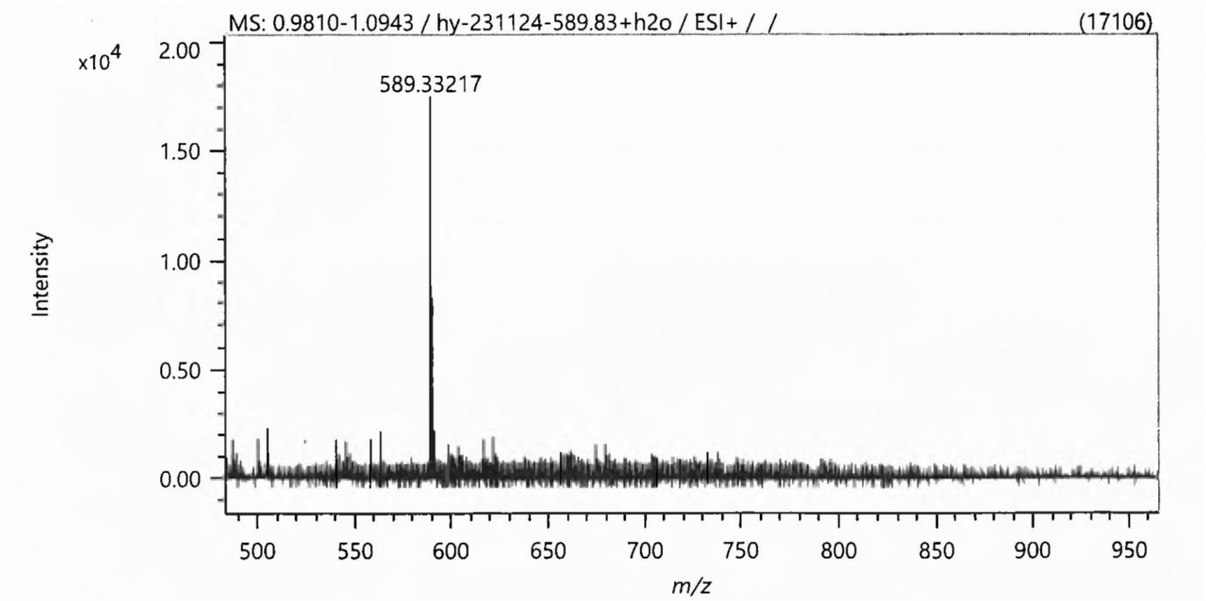

Figure S3. Mass spectra of **TPAP1**.

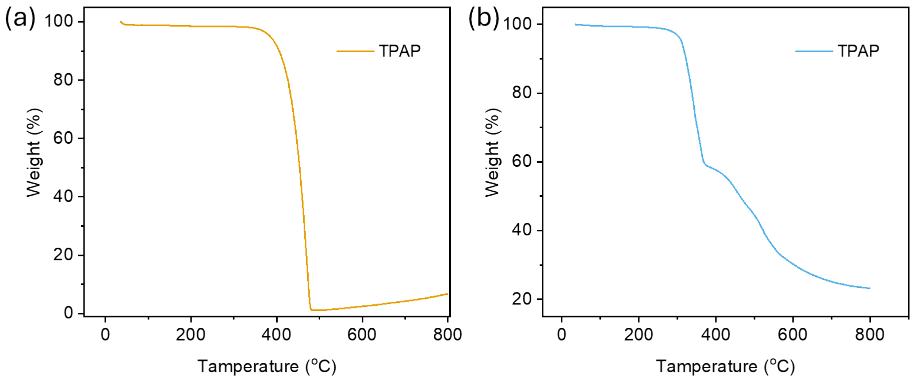

Figure S4. Thermogravimetric analysis (TGA) of (a) **TPAP** and (b) **TPAP1**.

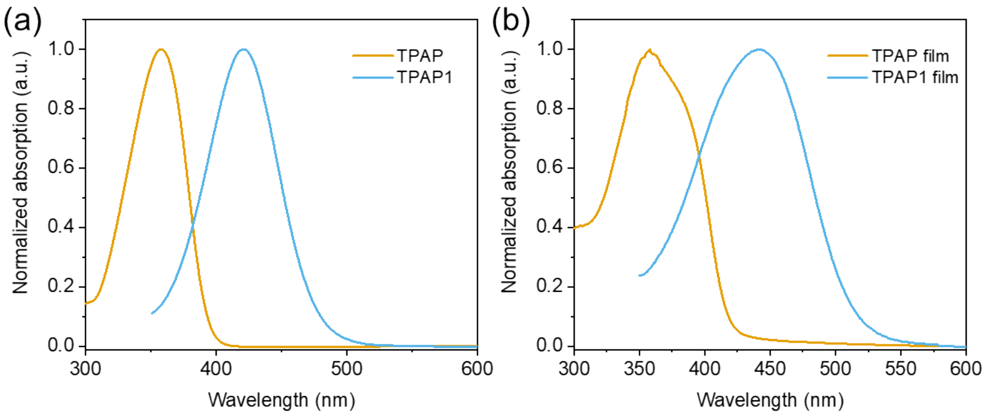

Figure S5. The UV-Vis spectra of **TPAP** and **TPAP1** (a) in DMF (b) as thin films.

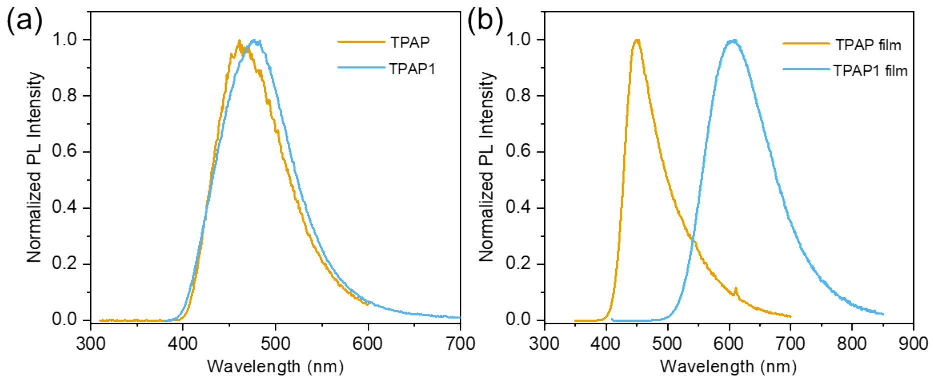

Figure S6. The PL spectra of **TPAP** and **TPAP1** (a) in DMF (b) as thin films.

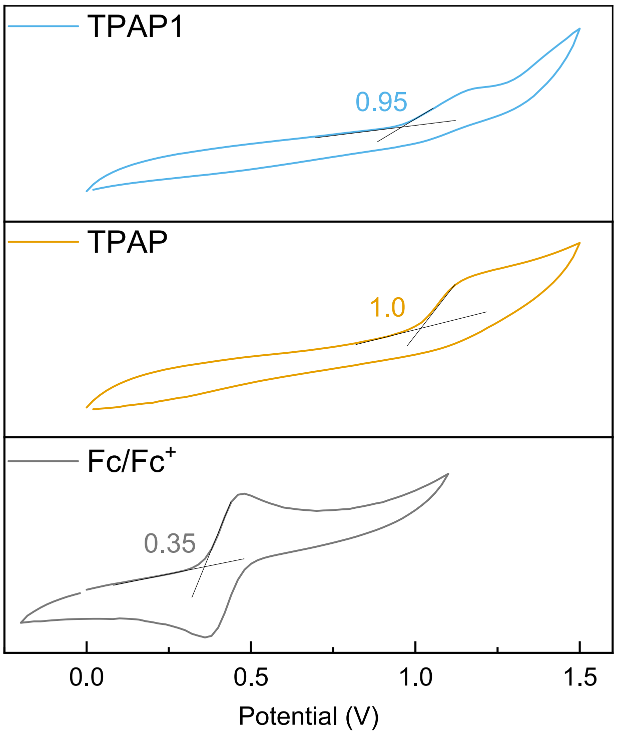

Figure S7. Cyclic voltammograms of **TPAP** and **TPAP1** measured in DMF with 0.1 TBAPF6 as electrolyte (scan rate = 100 mV s^{-1}).

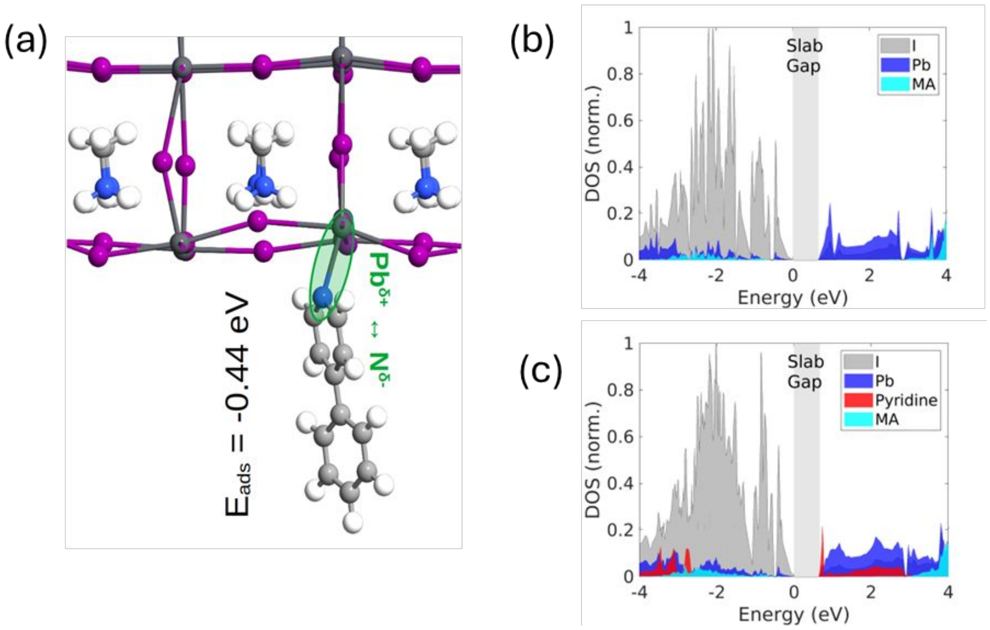

Figure S8. a) Optimized configuration for the unmodified pyridine group of **TPAP** interacting with the PbI_2 terminated perovskite surface, where Pb, I, N, C, and H atoms are represented in dark gray, purple, blue, gray, and white, respectively. The key interaction is highlighted by the shaded area, and the obtained adsorption energy is reported. b) PDOS of the free-standing perovskite slab. c) PDOS for the interacting system (perovskite surface + pyridine group of **TPAP**).

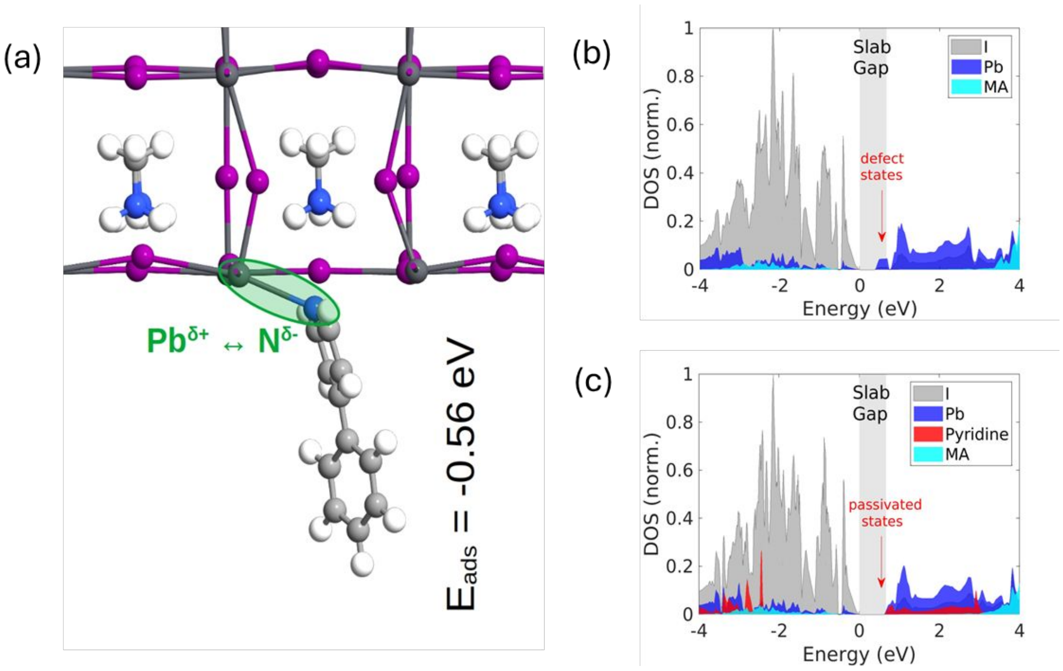

Figure S9. a) Optimized configuration for the unmodified pyridine group of **TPAP** interacting with the perovskite surface with iodine vacancy, where Pb, N, C, and H atoms are represented in dark gray, purple, blue, gray, and white, respectively. The key interaction is highlighted by the shaded area, and the obtained adsorption energy is reported. b) PDOS of the free-standing

perovskite slab with V_I surface defects, where the gap of the perovskite slab without V_I defects is also reported for comparison. c) PDOS for the interacting system (perovskite surface with defects + pyridine group of **TPAP**).

In **Figure S8**, the optimized configuration for the unmodified pyridine group interacting with the PbI_2 terminated perovskite surface is reported along with the PDOS of the perovskite slab and of the interacting system. The negatively charged N atom on the pyridine group interacts with the under-coordinated $Pb^{\delta+}$ ion on the perovskite surface, with an adsorption energy of E_{ads}^{TPAP} = -0.44 eV, and no significant effects on the density of perovskite surface states. The results obtained when the presence of iodine vacancies on the perovskite surface is accounted for are represented in **Figure S9**. The nitrogen atom on the pyridine group, still interacting with the under-coordinated $Pb^{\delta+}$ on the perovskite surface, moves towards the position of the missing iodine atom and passivates the defect states, resulting in an adsorption energy of only E_{ads}^{TPAP} = -0.56 eV, though.

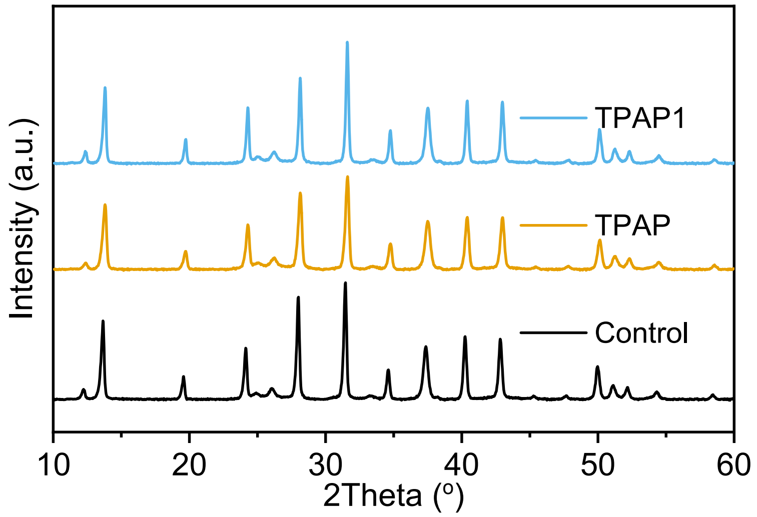

Figure S10. XRD spectra of perovskite films without and with **TPAP** and **TPAP1**.

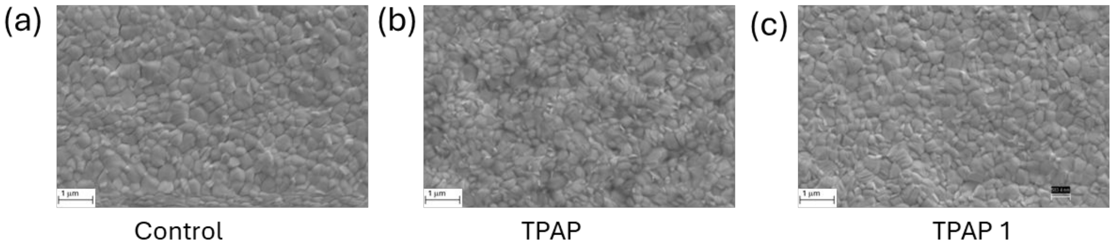

Figure S11. Surface SEM-images of perovskite films (a) without and with (b) **TPAP** and (c) **TPAP1**.

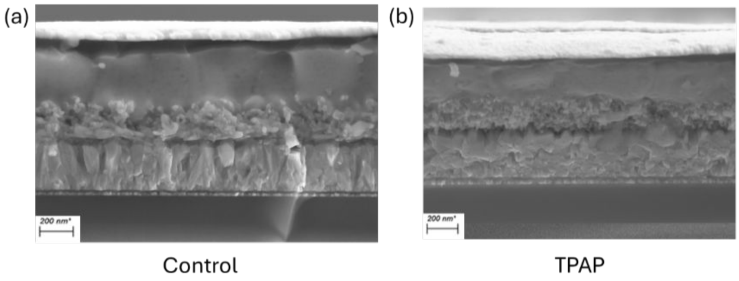

Figure S12. Cross-sectional SEM-images of perovskite solar cells (a) without and with (b) **TPAP**.

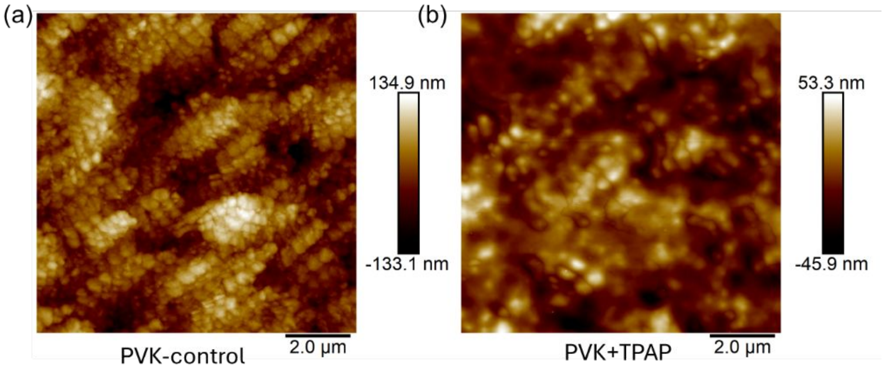

Figure S13. Topography images obtained using atomic force microscopy of (a) bare perovskite, (b) perovskite/**TPAP**.

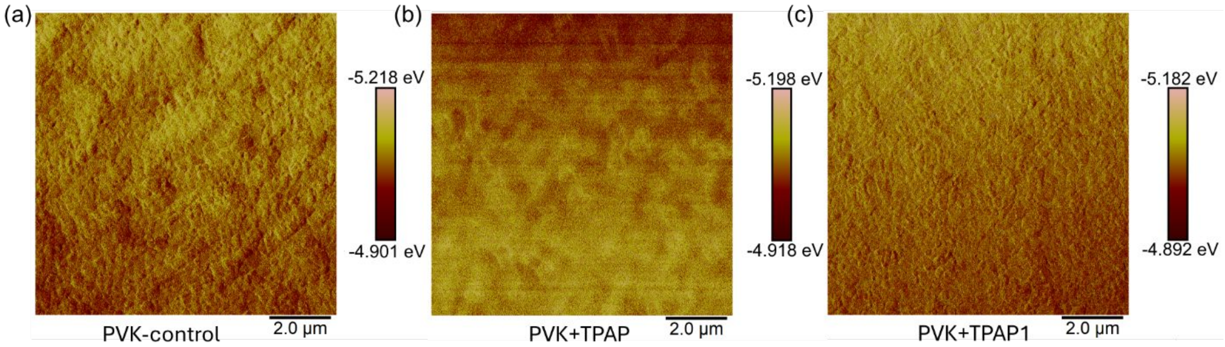

Figure S14. Kelvin probe force microscopy images of (a) bare perovskite, (b) perovskite/**TPAP** and c) perovskite/**TPAP1**.

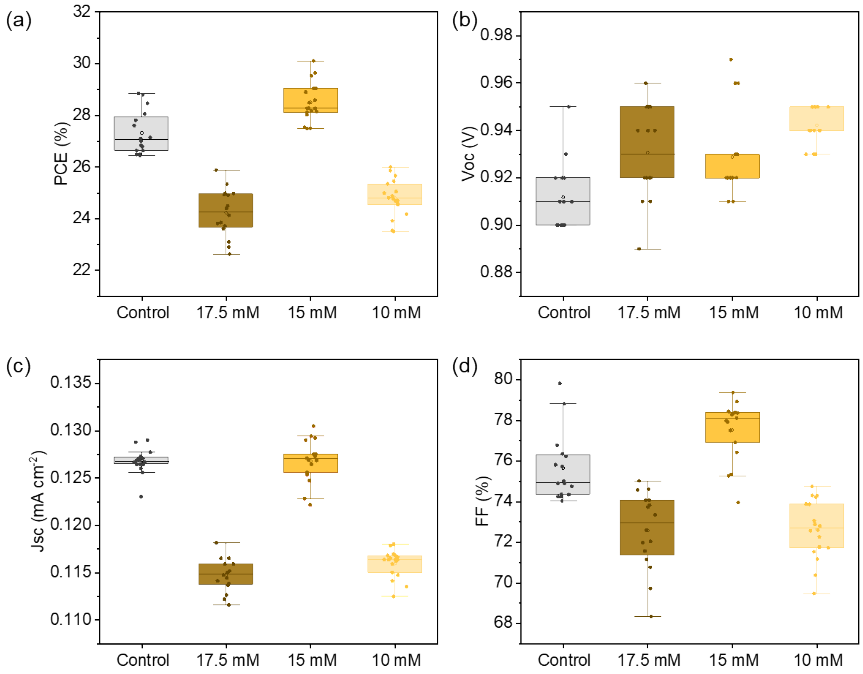

Figure S15. Performance of perovskite solar cells at different **TPAP** concentrations under 1000 lux WLED (4000 K) illumination. (a) PCE (b) V_{OC} (c) Jsc (d) FF.

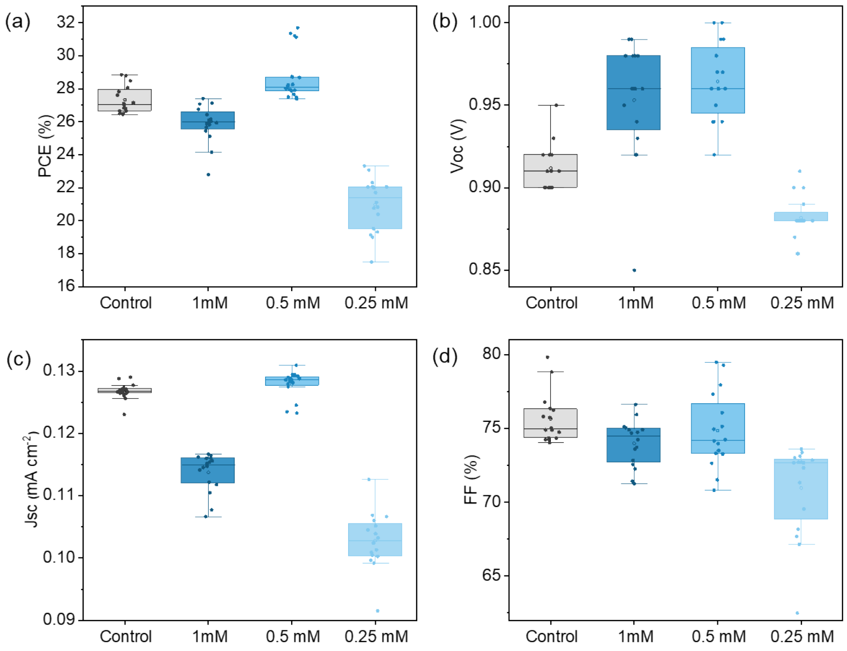

Figure S16. Performance of perovskite solar cells at different **TPAP1** concentrations under 1000 lux WLED (4000 K) illumination. (a) PCE (b) V_{OC} (c) Jsc (d) FF.

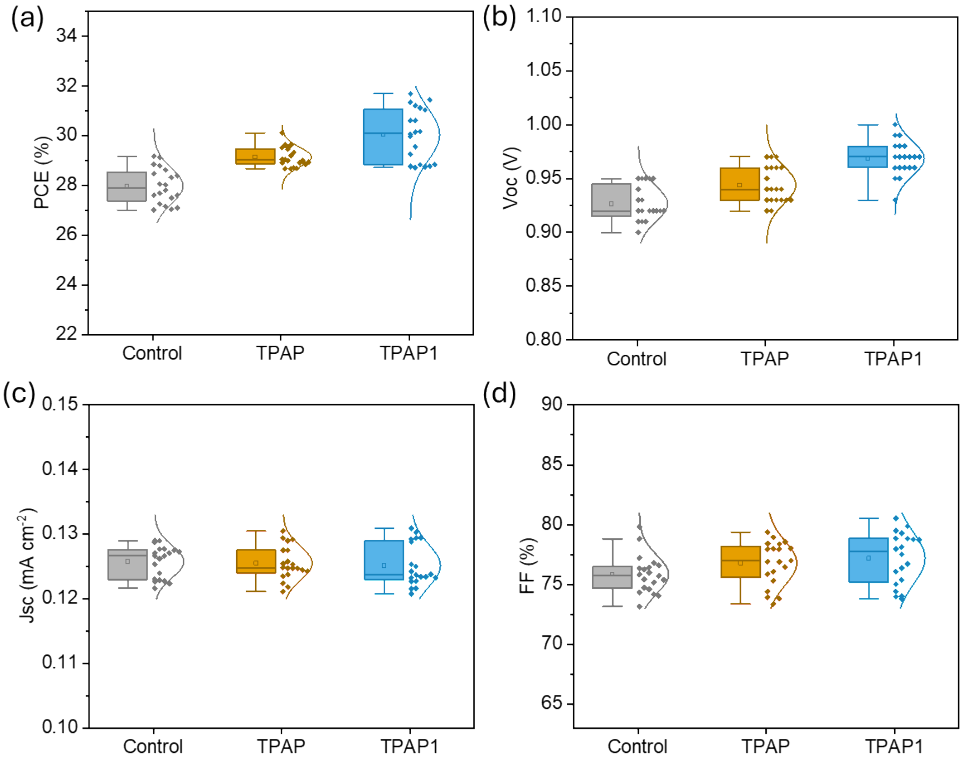

Figure S17. Statistical representation of the photovoltaic parameters derived from the reverse scans of 20 PSCs for each condition under 1000 lux WLED (4000 K) illumination (with a scan rate of 100 mV s^{-1}). (a) PCE (b) V_{OC} (c) Jsc (d) FF.

Table S1. Reported indoor efficiency of perovskite solar cells based on different bandgaps perovskites under different illumination conditions.

Perovskite composition	Band gap (eV)	Modification	Illumination conditions	PCE(i)	Voc	REF.
$(FAPbI_3)_{0.97}(MAPbBr_3)_{0.03}$	1.59	Surface treatment	LED 2700 K, 824.5 lux	40.10%	1.00	1
Single-Crystal $MAPbI_3$	1.5	HTL	LED 2700 K, 1000 lux	39.20%	0.93	2
$Cs_{0.15}FA_{0.85}PbI_3$	1.48	Surface treatment	LED 6500 K, 1000 lux	39.04%	0.93	3
$Rb_{0.05}Cs_{0.05}[(FA_{0.83}MA_{0.17})]_{0.9}Pb(I_{0.83}Br_{0.17})_3$	1.61	Interface Engineering	LED 3376 K, 984 lux	41.04%	1.08	4
$FA_{0.085}MA_{0.1}5PbI_3$	1.54	Additive	LED 3000 K, 1000.52 lux	42.12%	0.984	5
$CH_3NH_3PbI_3$	1.55	Interface Engineering	LED 6500 K, 1000 lux	34.51%	0.92	6
$MAPbI_3$	1.58	Surface treatment	LED 5000 K, 1000 lux	37.58%	0.936	7
**$Cs_{0.05}(MA_{0.17}FA_{0.83})_{0.95}Pb(I_{0.83}Br_{0.17})_3$**	**1.6**	**Surface treatment**	**LED 4000 K, 1000 lux**	**31.7%**	**1.00**	**This work**

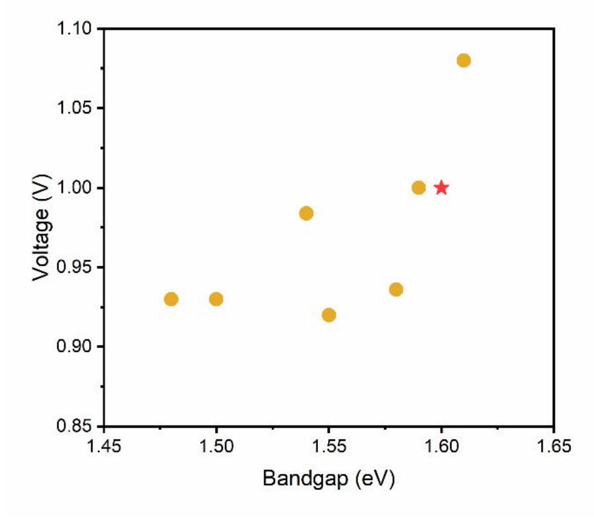

Figure S18. The corresponding V_{OC} versus bandgap of the indoor perovskite solar cells listed in Table S1.[1-7]

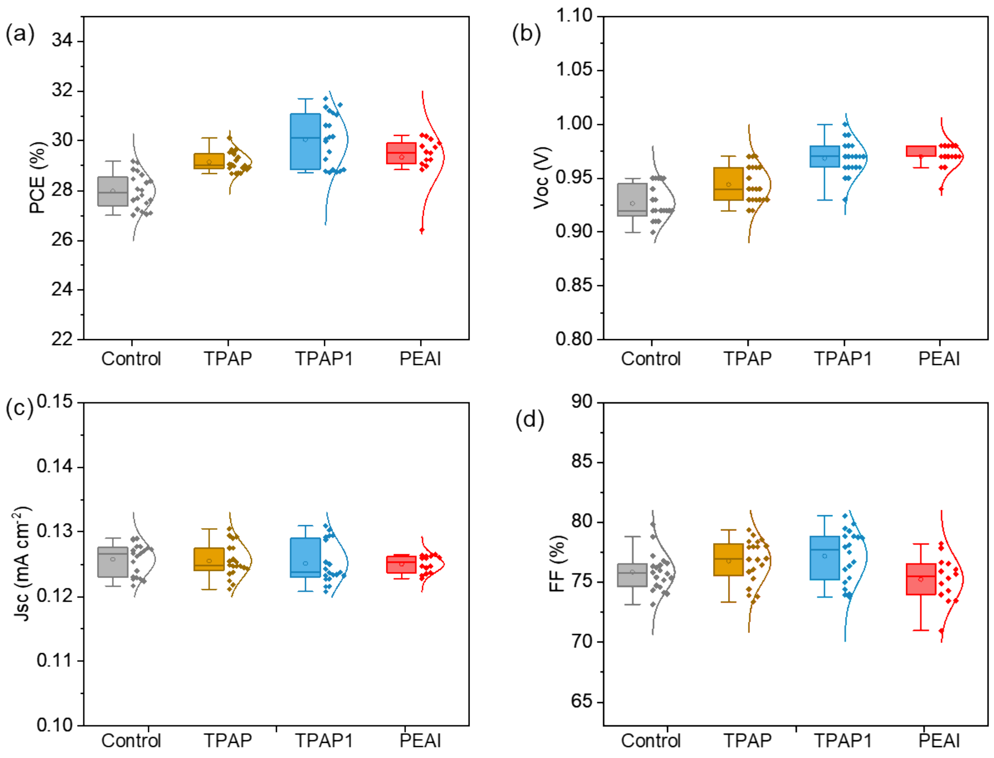

Figure S19. Photovoltaic performance comparison of **TPAP**, **TPAP1** and **PEAI** derived from the reverse scans of 20 PSCs for each condition under 1000 lux WLED (4000 K) illumination. (a) PCE (b) V_{OC} (c) Jsc (d) FF.

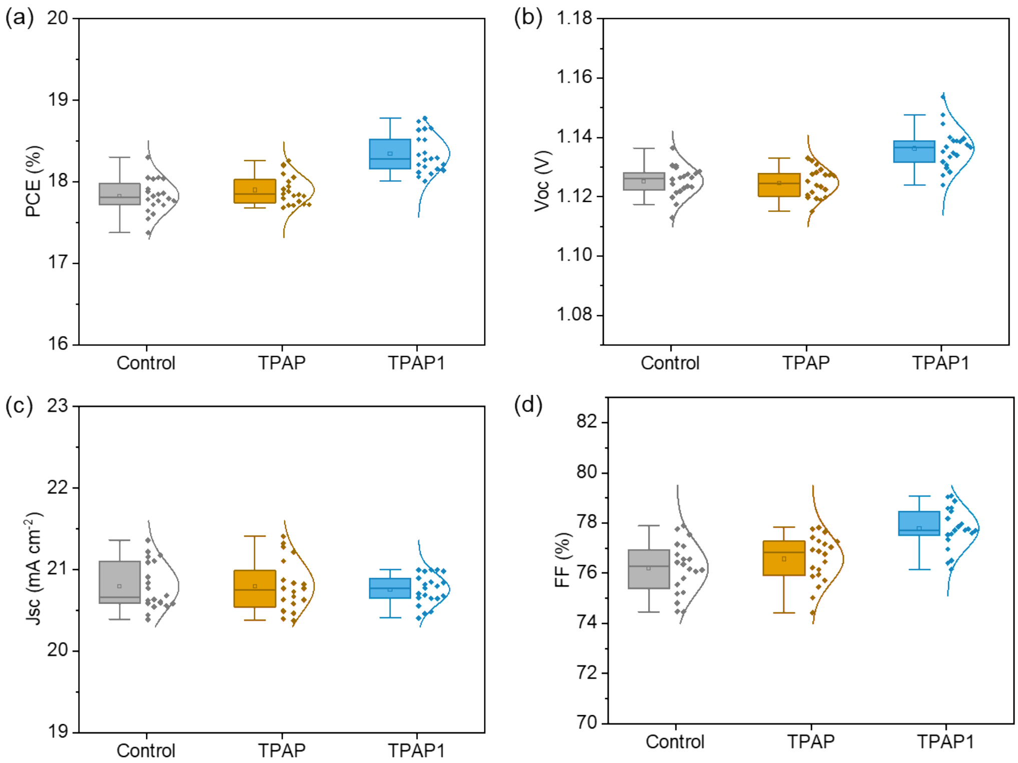

Figure S20. Statistical representation of the photovoltaic parameters derived from the reverse scans of 20 PSCs for each condition under 1-Sun illuminations (with a scan rate of 100 mV s^{-1}). (a) PCE (b) V_{OC} (c) Jsc (d) FF.

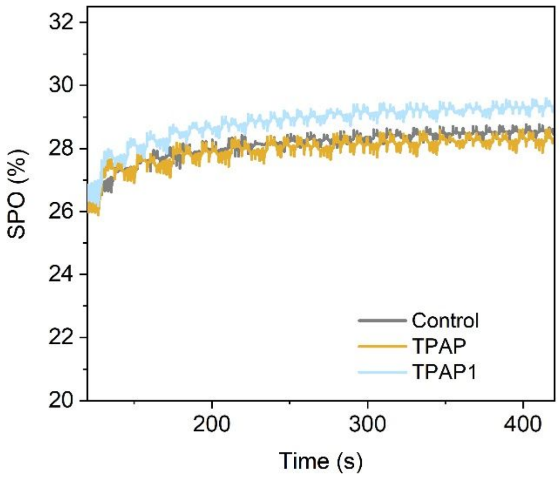

Figure S21. Stable power output (SPOs) at bias voltages perovskite devices with or without passivators at 1000 lux.

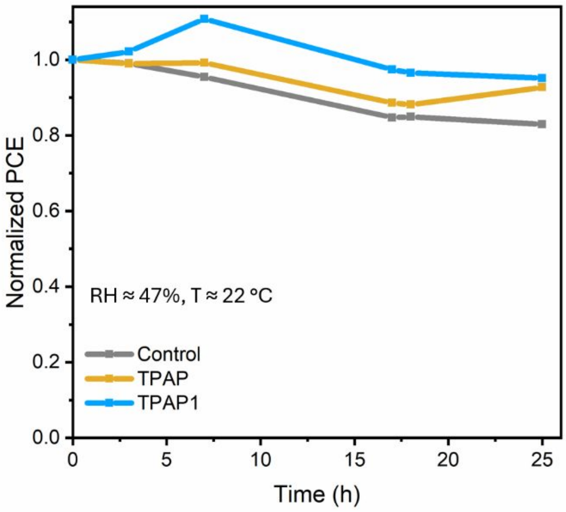

Figure S22. Normalized PCE of the best-performing **TPAP**-passivated and control devices under 1-sun illumination in ambient air (RH ≈ 47%, T ≈ 22 °C).

Table S2. Decay curve parameters fitted to time-resolved photoluminescence results.

Sample	A1 (%)	τ1 (ns)	A2 (%)	τ2 (ns)	A3 (%)	τ3 (ns)	τ_{avg} (ns)
Control	0.0708	2334	1.55	601	5.88	190.9	522
TPAP	0.0893	3202	1.76	736	5.817	222.8	749.4
TPAP1	0.1225	3970	1.851	940	5.772	260.1	1059

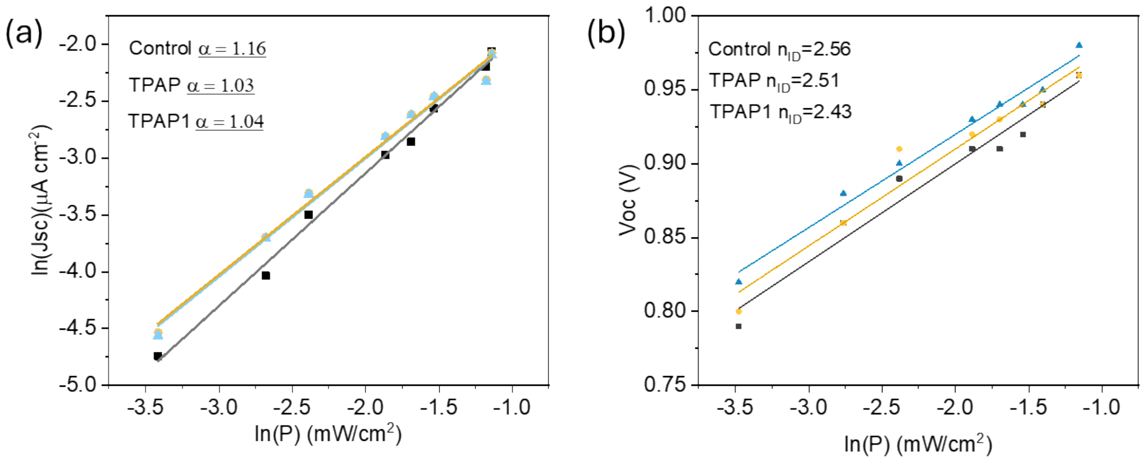

Figure S23. (a) J_{SC}- and (b) V_{OC}- light intensity plots of perovskite films with and without passivators.

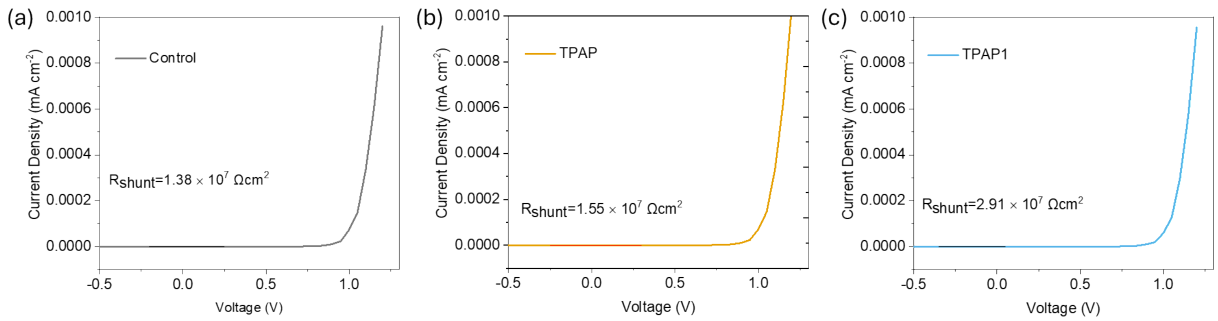

Figure S24. Shunt resistance extraction from a dark *J–V* curve (a) control (b) **TPAP** (c) **TPAP1**.

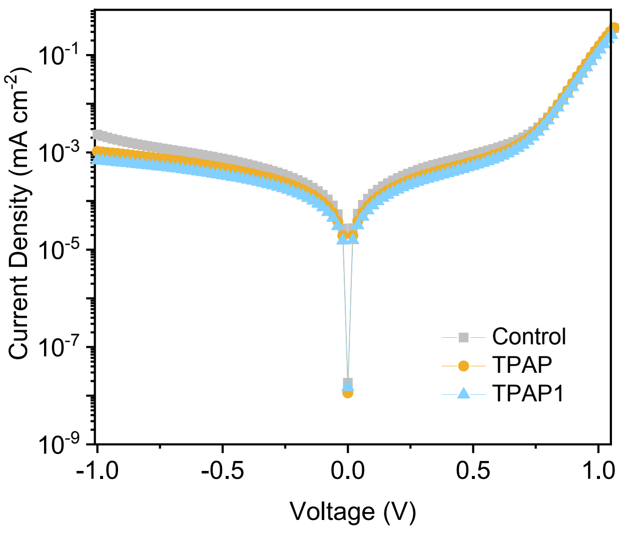

Figure S25. Current leakage extraction from a dark *J–V* curve with and without passivators.

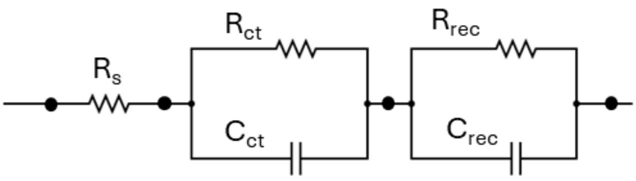

Figure S26. The equivalent circuit model used for fitting the EIS data of the perovskite solar cells.

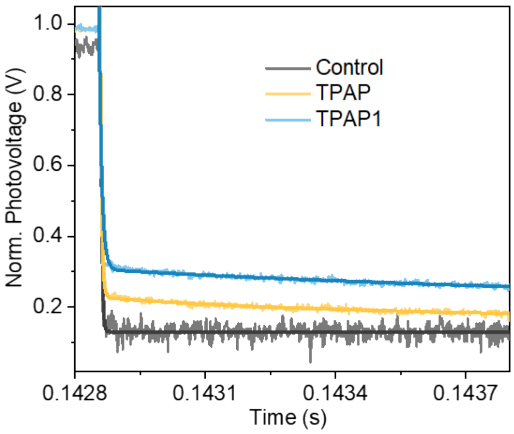

Figure S27. TPV decays of perovskite devices with or without passivators under a low-frequency light source.

Table S3. TPV decay curve parameters with or without passivators.

Sample	T1 (s)	T2 (s)	τavg (μs)
TPAP1	1.84×10^{-6}	3.19×10^{-5}	24.9
TPAP	1.09×10^{-6}	2.52×10^{-5}	4.91
Control	1.06×10^{-6}	2.45×10^{-5}	3.72

References

(1) He, X.; Chen, J.; Ren, X.; Zhang, L.; Liu, Y.; Feng, J.; Fang, J.; Zhao, K.; Liu, S. 40.1% Record Low-Light Solar-Cell Efficiency by Holistic Trap-Passivation using Micrometer-Thick Perovskite Film. *Adv. Mater.* **2021**, *33* (27), 2100770.
(2) Li, N.; Feng, A.; Guo, X.; Wu, J.; Xie, S.; Lin, Q.; Jiang, X.; Liu, Y.; Chen, Z.; Tao, X. Engineering the Hole Extraction Interface Enables Single-Crystal MAPbI3 Perovskite Solar Cells with Efficiency Exceeding 22% and Superior Indoor Response. *Adv. Energy Mater.* **2022**, *12* (7), 2103241.
(3) Lee, Y. S.; Jae Do, J.; Jung, J. W. A comparative study of surface passivation of p-i-n perovskite solar cells by phenethylammonium iodide and 4-fluorophenethylammonium iodide for efficient and practical perovskite solar cells with long-term reliability. *J. Alloys Compd.* **2024**, *988*, 174060.
(4) Li, C.; Sun, H.; Dou, D.; Gan, S.; Li, L. Bipolar Pseudohalide Ammonium Salts Bridged Perovskite Buried Interface toward Efficient Indoor Photovoltaics. *Adv. Energy Mater.* **2024**, *14* (34), 2401883.
(5) Li, Y.; Nie, T.; Ren, X.; Wu, Y.; Zhang, J.; Zhao, P.; Yao, Y.; Liu, Y.; Feng, J.; Zhao, K.; et al. In Situ Formation of 2D Perovskite Seeding for Record-Efficiency Indoor Perovskite Photovoltaic Devices. *Adv. Mater.* **2024**, *36* (1), 2306870.
(6) Shin, S. J.; Alosaimi, G.; Choi, M. J.; Ann, M. H.; Jeon, G. G.; Seidel, J.; Kim, J.; Yun, J. S.; Kim, J. H. Strategic Approach for Frustrating Charge Recombination of Perovskite Solar Cells in Low-Intensity Indoor Light: Insertion of Polar Small Molecules at the Interface of the Electron Transport Layer. *ACS Appl. Energy Mater.* **2022**, *5* (11), 13234-13242.
(7) Sandhu, S.; Rahman, M. M.; Yadagiri, B.; Kaliamurthy, A. K.; Mensah, A. E.; Lima, F. J.; Ahmed, S.; Park, J.; Kumar, M.; Lee, J.-J. Surface Reconstruction with Aprotic Trimethylsulfonium Iodide for Efficient and Stable Perovskite Solar Cells. *ACS Appl. Mater. Interfaces* **2024**, *16* (3), 4169-4180.
